# Supplementary material for: The relationship between income poverty and child hospitalisations in New Zealand: Evidence from longitudinal household panel data and Census data
Source: PLoS One. 2021 Jan 13;16(1):e0243920. doi: 10.1371/journal.pone.0243920 (PMC7806187; doi:10.1371/journal.pone.0243920)
Supplement: S1 Appendix — (DOCX) [file pone.0243920.s001.docx]

S1 Appendix. International Statistical Classification of Diseases and Related Health Problems 10th Revision (ICD-10) codes used for hospitalisations

1. Otitis media: H65 - H67, H72

2. Oral health: K02, K04, K05

3. Infectious disease, including:

- enteric infections: A00 – A08
- enteric symptoms: A09, I88, K52.8, K52.9, R11
- septicaemia: A40, A41
- sexually transmitted infections: A50 – A60, A63, A64, N29
- HIV/AIDS: B20-B24
- Meningococcal disease: A39
- Central nervous system (CNS) viral infections: A80.1 – A80.4, A80.9, A81.1 – A81.2, A81.8 – A81.9, A82 – A89
- CNS general infections: G00 – G03.0, G03.9, G04 – G09, G61.0
- Eye infections: B30, H00.0, H03, H04.3, H05.0, H10.0, H10.2, H10.3, H10.9, H13.0, H13.1, H16.0, H19.0 - H19.2, H22.0, H44.0, H45.1
- Ear infections: H60.0 – H60.3, H60.8, H60.9, H62, H65 – H67, H68.0, H70, H73.0, H75.0, H83.0, H94.0
- Upper respiratory tract infections (RTIs): J00 - J06, J32, J34.0, J36, J37, J39.0, J39.1
- Tuberculosis: A15 - A19, N74.0, N74.1, J65
- Acute lower RTIs: A48.1, A48.2, B59, J09 - J18, J20 - J22
- Chronic lower RTIs: J40 - J42, J44.0, J47, J85, J86, J98.8
- Heart and circulatory infections: B33.2, I00 - I02, I05 - I09, I30.1, I33, I38, I39, I40.0, I41.0 - I41.2 I43.0, I71.6, I79.0, I79.1
- Oral infections: K02, K04.4, K04.6, K05.0, K05.2, K05.3, K11.3, K12.2
- Gastrointestinal tract infections: K23.0, K23.1, K25 - K28, K29.3 - K29.5, K35 - K37, K61, K63.0, K63.2, K65.0, K67.8, K90.8, K93.0
- Hepatic infections: K75.0, K77.0, K83.0
- Viral hepatitis: B15-B19
- Kidney infections: N00, N05, N10, N13.6, N15.1
- Urinary tract infection: N30.0, N34.1, N35.1, N37, N39.0
- Reproductive system infections, male: N41.0 - N41.3, N43.1, N45, N48.1 - N48.2, N49, N51
- Reproductive system infections, female: N70 - N73, N75.0 - N75.1, N76.4, N87
- Skin infections, typical: A46, L00 - L04, L05.0, L08
- Skin infections, other: B86, S10.13, S10.83, S10.93, S20.13, S20.33, S20.43, S20.83, S30.83, S30.93, S40.83, S50.83, S60.83, S70.83, S80.83, S90.83, T0.09, T09.03, T11.08, T13.03, T14.03, T63.3, T63.4, T79.3, T89.01, T89.02
- Breast infections: N61
- Osteomyelitis: M46.2 - M46.5
- Joint infections: M00 – M01
- Connective tissue infections: M02.1, M02.3, M03, M60.0, M63.0 - M63.2, M65.0 - M65.1, M68.0, M71.0 - M71.1, M89.6
- Neoplasms from infection: C11, C16.1 - C16.6, C16.8, C16.9, C21.0, C21.1, C22.0, C46, C53, D00.2, D01.3, D06
- Postoperative infections: T80.2, T81.4, T82.6, T82.7, T83.5, T83.6, T84.5 - T84.7, T85.7, T87.4
- Adverse effect of Infectious Disease Treatment: R76.1 - R76.2, T36, T37, T48.5, T48.7, T49.0, T49.5, T49.6, T49.9, T78.8 - T78.9, T88.0 - T88.1, T88.7
- Other Bacterial infections: A20 - A28, A30 - A38, A42 - A44, A48.0, A48.3, A48.4, A48.8, A49, A65 - A71, A74, A75, A77 - A79, B95 - B96
- Other Viral infections: A90 - A96, A98 - A99, B00 - B07, B09, B25 - B27, B33.0, B33.1, B33.3 - B34, B97
- Other Mycoses: B35 – B49
- Other Protozoan infections: B50 - B58, B60, B64
- Other infectious diseases: B65 - B83, B85, B87 - B89, B94, B99, E033, E32.1, F02.4, F071, I88.1 - I88.9, T64

4. Respiratory condition: A15 - A19, J00 - J06, J10 - J16, J18, J21, J45 - J47

5. Preventable hospitalisation, including:

- Acute bronchiolitis: J21
- I00 - I02Acute rheumatic fever: I00 - I02
- Acute upper respiratory tract infection excluding croup: J00 - J03, J06
- Asthma: J45, J46
- Bronchiectasis: J47
- Bacterial meningitis: G00, G01
- Bacterial/ Unspecified pneumonia: J13 - J16, J18
- Constipation: K59.0
- Chronic rheumatic heart disease: I05 - I09
- Croup, acute laryngitis, tracheitis: J04, J05.0
- Dental (dental caries, pulp, periodontal): K02, K04, K05
- Dermatitis/eczema: L20 - L30
- Febrile convulsions: R560
- Gastroenteritis: A00 - A09, R11, K52.9
- Gastro oesophageal reflux: K21
- Meningococcal disease: A39
- Nutritional deficiency: E40 - E64, D50 - D53
- Otitis media: H65 - H67
- Osteomyelitis: M86
- Vaccine preventable diseases: P35.0, A33 - A37, A80, B16, B18.0 - B18.1, B05 - B06, B26, M01.4
- Skin infection: L00 - L05, L08, L98.0, J34.0, H01.0, H00.0
- Tuberculosis: A15 - A19
- Urinary tract infection: N10, N12, N30.0, N39.0, N13.6, N30.9
- Viral pneumonia: J12, J10.0, J11.0
- Viral / other / unspecified meningitis: A87,G02 - G03
- Viral infection of unspecified site: B34
