# Supplementary material for: The relationship between income poverty and child hospitalisations in New Zealand: Evidence from longitudinal household panel data and Census data
Source: PLoS One. 2021 Jan 13;16(1):e0243920. doi: 10.1371/journal.pone.0243920 (PMC7806187; doi:10.1371/journal.pone.0243920)
Supplement: S2 Appendix — (DOCX) [file pone.0243920.s002.docx]

S2 Appendix. Association between household equivalised income and hospitalisations using local polynomial regressions.

The figures below show the relationship between a) self-reported BHC income and b) tax-reported BHC income and each hospitalisation outcome using SoFIE data. These are estimated using a local polynomial regression, which can be described intuitively as a smoothed out scatter plot. This allows us to visualise the relationship without assuming a specific functional form (such as linear). Local polynomial regression involves fitting the response to a polynomial form of the regressor via locally weighted least squares. Local regression means that only data points in the ‘neighbourhood’ of a given X value are used and data points that are closer on the X axis are given a higher weight. The size of the neighbourhood, and hence the degree of smoothing, is controlled by choosing a bandwidth. The appropriate bandwidth was estimated using the Rule of Thumb (ROT) estimator, a plug-in estimator of the asymptotically optimal constant bandwidth, which minimizes the conditional weighted mean integrated squared error.

For the figures below income is equivalised and trimmed and presented as percentiles for ease of interpretation. Note that the relationship looks similar for the association between disposable income (i.e., self-reported income less tax and housing costs) and hospitalisation outcomes. These are not shown below but are available on request.

Figure 1. Local polynomial smooth showing the association between a) self-report and b) tax-recorded household equivalised income before housing costs (BHC) and hospitalisation for Otitis Media.

1. Self-Reported BHC income

Proportion hospitalised for Otitis Media

1. Tax-recorded BHC income

Percentile of equivalised household income

Proportion hospitalised for Otitis Media

Figure 2. Local polynomial smooth showing the association between a) self-report and b) tax-recorded household equivalised income before housing costs (BHC) and hospitalisation for oral health.

1. Self Reported BHC income

Proportion hospitalised for oral health

1. Tax-recorded BHC income

Percentile of equivalised household income

Proportion hospitalised

Proportion hospitalised for oral health

Figure 3. Local polynomial smooth showing the association between a) self-report and b) tax-recorded household equivalised income before housing costs (BHC) and hospitalisation for respiratory conditions.

1. Self Reported BHC income

Proportion hospitalised for Respiratory Conditions

1. Tax-recorded BHC income

Percentile of equivalised household income

Proportion hospitalised for Respiratory Conditions

Figure 4. Local polynomial smooth showing the association between a) self-report and b) tax-recorded household equivalised income before housing costs (BHC) and hospitalisation for Infectious diseases.

1. Self Reported BHC income

Proportion hospitalised for Infectious Diseases

1. Tax-recorded BHC income

Percentile of equivalised household income

Proportion hospitalised for Infectious Diseases

Figure 5. Local polynomial smooth showing the association between a) self-report and b) tax-recorded household equivalised income before housing costs (BHC) and hospitalisation for Preventable Admissions.

1. Self Reported BHC income

Proportion hospitalised for Preventable Admissions

1. Tax-recorded BHC income

Proportion hospitalised for Preventable Admissions

Percentile of equivalised household income

Figure 6. Local polynomial smooth showing the association between a) self-report and b) tax-recorded household equivalised income before housing costs (BHC) and any admission to hospital.

1. Self Reported income

Proportion hospitalised for any Admission

1. Tax-recorded income

Proportion hospitalised for any Admission

Percentile of equivalised household income
