# Supplementary material for: The relationship between income poverty and child hospitalisations in New Zealand: Evidence from longitudinal household panel data and Census data
Source: PLoS One. 2021 Jan 13;16(1):e0243920. doi: 10.1371/journal.pone.0243920 (PMC7806187; doi:10.1371/journal.pone.0243920)
Supplement: S1 Table — (DOCX) [file pone.0243920.s003.docx]

S1 Table. Association between income poverty and hospitalisations stratified by wave for SoFIE children

|  | Otitis Media | Oral Health | Infectious | Respiratory | Preventable | Any admission |
| --- | --- | --- | --- | --- | --- | --- |
| **Wave stratification** |  |  |  |  |  |  |
| BHC – self report |  |  |  |  |  |  |
| Wave 1 | 0.78(0.40;1.52) | 1.21(0.60;2.40) | 1.08(0.83;1.41) | 1.25(0.78;2.02) | 1.02(0.79;1.32) | 1.17(0.98;1.39) |
| Wave 2 | 1.60(0.93;2.75) | 1.11(0.49;2.48) | 1.13(0.84;1.54) | 0.85(0.47;1.54) | 1.28(0.96;1.71) | 1.18(0.97;1.43) |
| Wave 7 | 2.10(0.85;5.11) | 1.15(0.35;3.83) | 1.32(0.91;1.92) | 1.15(0.60;2.24) | 1.18(0.81;1.74) | 0.95(0.75;1.20) |
| Wave 8 | 1.35(0.54;3.43) | 1.16(0.44;3.09) | 1.15(0.78;1.68) | 1.20(0.62;2.35) | 1.03(0.70;1.52) | 1.14(0.91;1.43) |
| BHC – tax records |  |  |  |  |  |  |
| Wave 1 | 1.01(0.53;1.95) | 1.53(0.77;3.01) | 1.26(0.97;1.64) | 1.32(0.81;2.14) | 1.21(0.93;1.57) | 1.12(0.94;1.34) |
| Wave 2 | 0.99(0.55;1.79) | 0.59(0.22;1.57) | 0.94(0.68;1.29) | 1.02(0.57;1.84) | 1.07(0.79;1.45) | 0.94(0.77;1.16) |
| Wave 7 | 1.94(0.79;4.77) | 2.16(0.69;6.68) | 1.27(0.88;1.84) | 1.21(0.63;2.32) | 1.21(0.83;1.76) | 0.93(0.74;1.18) |
| Wave 8 | 0.82(0.29;2.26) | 1.60(0.61;4.19) | 0.89(0.60;1.33) | 1.05(0.53;2.08) | 1.03(0.70;1.51) | 1.08(0.86;1.31) |
| AHC – self report |  |  |  |  |  |  |
| Wave 1 | 1.19(0.65;2.19) | 1.25(0.64;2.46) | 1.17(0.91;1.52) | 1.45(0.91;2.33) | 1.14(0.88;1.46) | 1.23(1.04;1.45) |
| Wave 2 | 1.67(0.99;2.88) | 1.01(0.46;2.23) | 1.25(0.94;1.68) | 1.10(0.63;1.91) | 1.44(1.09;1.91) | 1.18(0.98;1.43) |
| Wave 7 | 3.03(1.19;7.69) | 2.48(0.78;7.79) | 1.60(1.11;2.28) | 1.10(0.58;2.10) | 1.36(0.95;1.96) | 1.06(0.85;1.33) |
| Wave 8 | 1.00(0.39;3.54) | 1.38(0.54;3.48) | 1.15(0.79;1.66) | 1.25(0.66;2.38) | 1.11(0.77;1.61) | 1.22(0.98;1.51) |

Note: interactions between age*poverty and wave*poverty were formally tested in logistic regression models for each hospitalisation outcome. In all instances p>0.05.
