# Supplementary material for: The relationship between income poverty and child hospitalisations in New Zealand: Evidence from longitudinal household panel data and Census data
Source: PLoS One. 2021 Jan 13;16(1):e0243920. doi: 10.1371/journal.pone.0243920 (PMC7806187; doi:10.1371/journal.pone.0243920)
Supplement: S2 Table — (DOCX) [file pone.0243920.s004.docx]

S2 Table. Association between quintiles of BHC self-reported income in SoFIE averaged over two waves with hospitalisations

|  |  |  |  |  | 95% Confidence Intervals | |
| --- | --- | --- | --- | --- | --- | --- |
| Otitis Media | OR | SE | t | p | lb | ub |
| Quintile 1 (low income) | 1.85 | 0.51 | 2.22 | 0.03 | 1.07 | 3.18 |
| Quintile 2 | 2.15 | 0.58 | 2.83 | 0.01 | 1.27 | 3.65 |
| Quintile 3 | 2.40 | 0.64 | 3.30 | 0.00 | 1.43 | 4.04 |
| Quintile 4 | 0.80 | 0.27 | -0.67 | 0.51 | 0.41 | 1.54 |
| Quintile 5 | Ref |  |  |  |  |  |
| Oral Health |  |  |  |  |  |  |
| Quintile 1 (low income) | 2.92 | 0.97 | 3.20 | 0.00 | 1.52 | 5.61 |
| Quintile 2 | 2.92 | 0.97 | 3.20 | 0.00 | 1.52 | 5.61 |
| Quintile 3 | 2.75 | 0.93 | 3.00 | 0.00 | 1.42 | 5.32 |
| Quintile 4 | 2.25 | 0.78 | 2.34 | 0.02 | 1.14 | 4.44 |
| Quintile 5 | Ref |  |  |  |  |  |
| Infectious |  |  |  |  |  |  |
| Quintile 1 (low income) | 1.43 | 0.18 | 2.84 | 0.00 | 1.12 | 1.84 |
| Quintile 2 | 1.64 | 0.20 | 4.04 | 0.00 | 1.29 | 2.09 |
| Quintile 3 | 1.78 | 0.22 | 4.75 | 0.00 | 1.40 | 2.26 |
| Quintile 4 | 1.12 | 0.15 | 0.82 | 0.42 | 0.86 | 1.45 |
| Quintile 5 | Ref |  |  |  |  |  |
| Respiratory |  |  |  |  |  |  |
| Quintile 1 (low income) | 1.10 | 0.27 | 0.37 | 0.71 | 0.67 | 1.78 |
| Quintile 2 | 1.35 | 0.32 | 1.29 | 0.20 | 0.85 | 2.15 |
| Quintile 3 | 1.42 | 0.33 | 1.50 | 0.13 | 0.90 | 2.24 |
| Quintile 4 | 1.23 | 0.30 | 0.84 | 0.40 | 0.76 | 1.97 |
| Quintile 5 | Ref |  |  |  |  |  |
| Preventable |  |  |  |  |  |  |
| Quintile 1 (low income) | 1.67 | 0.22 | 3.93 | 0.00 | 1.29 | 2.15 |
| Quintile 2 | 1.87 | 0.24 | 4.93 | 0.00 | 1.46 | 2.40 |
| Quintile 3 | 1.89 | 0.24 | 5.03 | 0.00 | 1.48 | 2.43 |
| Quintile 4 | 1.37 | 0.18 | 2.30 | 0.02 | 1.05 | 1.78 |
| Quintile 5 | Ref |  |  |  |  |  |
| Any Admission |  |  |  |  |  |  |
| Quintile 1 (low income) | 1.09 | 0.08 | 1.22 | 0.22 | 0.95 | 1.27 |
| Quintile 2 | 1.31 | 0.09 | 3.79 | 0.00 | 1.14 | 1.50 |
| Quintile 3 | 1.34 | 0.09 | 4.10 | 0.00 | 1.16 | 1.53 |
| Quintile 4 | 1.09 | 0.08 | 1.10 | 0.27 | 0.94 | 1.26 |
| Quintile 5 | Ref |  |  |  |  |  |
