# Supplementary material for: The relationship between income poverty and child hospitalisations in New Zealand: Evidence from longitudinal household panel data and Census data
Source: PLoS One. 2021 Jan 13;16(1):e0243920. doi: 10.1371/journal.pone.0243920 (PMC7806187; doi:10.1371/journal.pone.0243920)
Supplement: Attachment — Submitted filename: Response to reviewers_update.docx [file pone.0243920.s005.docx]

Barry Milne

COMPASS Research Centre

The University of Auckland

Auckland

New Zealand

[b.milne@auckland.ac.nz](mailto:b.milne@auckland.ac.nz)

Dear Professor Picone,

We thank the Associate Editor for the opportunity to revise our manuscript, and for the useful comments made by the reviewer. These comments are addressed below. All page and line numbers refer to the ‘Manuscript’ file.

Associate Editor comments

*In my opinion, the main concern is that the manuscript must address the endogeneity of income poverty.*

This has been addressed as described in our response to comment 1 of reviewer 1 below, and is described on p4-6 of the revised manuscript.

*Also, the manuscript should explore the mechanisms by which poverty affects hospitalizations.*

This has been addressed as described in our response to comment 2 of reviewer 1 below, and in the additional analyses we describe and present at the end of this document. We also describe these mechanisms on p3-4 of the revised manuscript.

*1. Please ensure that your manuscript meets PLOS ONE's style requirements, including those for file naming.*

We believe this has been done.

*2. Please provide additional details regarding participant consent. In the ethics statement in the Methods and online submission information, please ensure that you have specified what type you obtained (for instance, written or verbal, and if verbal, how it was documented and witnessed). If your study included minors, state whether you obtained consent from parents or guardians.*

*Once you have amended this/these statement(s) in the Methods section of the manuscript, please add the same text to the “Ethics Statement” field of the submission form (via “Edit Submission”).*

We have amended the ethics statement in the submission form, and in the methods section (lines 142-145):

“This study received ethics approval from the University of Auckland Human Ethics Committee (019618). Access to the anonymised data used in this study, including both SoFIE and Census data, was provided by Statistics NZ under the security and confidentiality provisions of the Statistics Act 1975.”

Also lines 153-156:

“Written consent was obtained from 79% adult participants to link to administrative health records^21^. Children were excluded from analyses if they had a consent-refusing parent at any wave, or if they refused consent themselves when aged 15 or over.”

Also, the Statistics New Zealand disclaimer regarding use of the data has been included after the acknowledgements, lines 532-552:

“Statistics New Zealand Disclaimer

The results in this paper are not official statistics. They have been created for research purposes from the Integrated Data Infrastructure (IDI), managed by Statistics New Zealand. The opinions, findings, recommendations, and conclusions expressed in this paper are those of the authors, not Statistics NZ. Access to the anonymised data used in this study was provided by Statistics NZ under the security and confidentiality provisions of the Statistics Act 1975. Only people authorised by the Statistics Act 1975 are allowed to see data about a particular person, household, business, or organisation, and the results in this paper have been confidentialised to protect these groups from identification and to keep their data safe. Careful consideration has been given to the privacy, security, and confidentiality issues associated with using administrative and survey data in the IDI. Further detail can be found in the Privacy impact assessment for the Integrated Data Infrastructure available from www.stats.govt.nz.

The results are based in part on tax data supplied by Inland Revenue to Statistics NZ under the Tax Administration Act 1994. This tax data must be used only for statistical purposes, and no individual information may be published or disclosed in any other form, or provided to Inland Revenue for administrative or regulatory purposes. Any person who has had access to the unit record data has certified that they have been shown, have read, and have understood section 81 of the Tax Administration Act 1994, which relates to secrecy. Any discussion of data limitations or weaknesses is in the context of using the IDI for statistical purposes, and is not related to the data’s ability to support Inland Revenue’s core operational requirements.”

*3.We note that you have indicated that data from this study are available upon request. PLOS only allows data to be available upon request if there are legal or ethical restrictions on sharing data publicly… a) If there are ethical or legal restrictions on sharing a de-identified data set, please explain them in detail (e.g., data contain potentially identifying or sensitive patient information) and who has imposed them (e.g., an ethics committee). Please also provide contact information for a data access committee, ethics committee, or other institutional body to which data requests may be sent.*

This is described in more detail below, and on the submission form. We also include text suggested by the editorial team to confirm that other researchers may apply for access to the dataset used in our study via Statistics New Zealand.

“Integrated Data Infrastructure (IDI) data cannot be shared publicly because of the security and confidentiality provisions of the Statistics Act 1975 (NZ). Data are held on servers maintained by Statistics New Zealand and are never released to researchers or posted on other sites. As such, the dataset is not owned or available to be distributed by the authors.

However, the authors had no special access privileges and other researchers are able to access the data in the same manner as the authors. Researchers who wish to access the data reported in the manuscript for analysis (or access IDI data generally) must submit an application through Statistics New Zealand (https://www.stats.govt.nz/integrated-data/integrated-data-infrastructure#how-apply). Data requests and queries about data access may be sent to access2microdata@stats.govt.nz.

If approved, data access is provided through a secure ‘Data Lab’ environment: a protected virtual environment in secure research facilities on computers that can access the IDI server, but nothing else (i.e. computer hard and soft drives cannot be accessed, and there is no access to the worldwide web). As such, IDI data are never sent to researchers or made available on data archives or via the worldwide web, but instead access is granted to analyse data within the Data Lab environment. Only the results of analyses (e.g., tables, models) can be requested to be released, and these must be confidentialised (e.g., all frequency counts are ‘random-rounded’ to be divisable by 3).

More details on IDI data access is provided in reference 18 of the manuscript (Milne et al., 2019) and here: <https://www.stats.govt.nz/integrated-data/integrated-data-infrastructure#data-safe>.”

Reviewer 1 comments

*1) In the introduction (from line 64), the authors mention that so far only very little evidence exists on the causal relationship between poverty and childhood health. Based on my interpretation of the study setup and the data used, I do not see how this paper is contributing to this. While the analysis uses high quality data and established research methods, the results only provide evidence for associations. As mentioned by the authors, this has been done by many others in the past. Thus, the authors need to make a better case for how their study contributes to the existing literature. Is using data from New Zealand one of the contributions or are there previous studies analysis the questions at hand with New Zealand data? I would recommend having a subsections highlighting the contributions of the study, especially given that these might be small overall.
From my understanding, previous studies have provided causal estimates by using exogenous variations in income as a result of changes in income assistance programs, for example.*

This paper contributes to the evidence base on the causal relationship between poverty and childhood health through our fixed-effects analyses (Table 5), which are considered by many to identify causal associations (under certain assumptions) by isolating whether within individual change in poverty status/income is associated with change in health (hospitalisations in our case). The systematic reviews we cite include only studies which “use methods that allow us to reach conclusions about causal relationships” (Cooper & Stewart, 2013; 2017), and specifically include studies with ‘fixed effects’ design as we use, as well as studies that use exogenous variations in income (as the reviewer highlights). In response to the reviewer we clarify our use of the word causal in the introduction on lines 86-90:

“Cooper and Stewart (2013; 2017)^10 16^ synthesised evidence from studies that attempt to isolate the causal effect of low income on child outcomes, either through quasi-experimental designs (e.g., programmes that increased incomes) or by statistical isolating the impact of low-income by analysing within household differences or within-individual change.”

We also describe in more detail the nature of the causal associations found – and not found in the Cooper and Stewart (2017) review on lines 94-100:

“There was evidence pointing to significant effects of exogenous changes in income (i.e., through payments or tax credits) on birthweight and other neonatal outcomes, as well as evidence that differences in household income experienced by children within families explained birthweight differences between children. However, there was mixed evidence for the impact of exogenous changes in income on obesity and general health in later childhood, and no evidence (from three studies) for an effect of exogenous changes in income on asthma, wheezing and other respiratory diseases^16^”

As suggested by the reviewer we also include a subsection at the end of the introduction (lines 105-122) which highlights the contribution of our study:

“This study contributes to the existing literature in the following ways. First, it represents an analysis of associations between poverty / low income and a range of hospitalisations thought to be poverty sensitive, specifically: respiratory conditions^19 20^, infectious diseases^8^, otitis media^18^, oral health^21 22^, and preventable admissions^23 24^. Second, we assess poverty in two samples: a longitudinal household panel survey: the Survey of Family, Income and Employment^25^; and whole population data from the 2013 New Zealand census; and we measure income from two sources: self-report and administrative tax records. Third, there have been debates regarding ‘sensitive periods’ for poverty experience, with some emphasising the importance of early childhood,^26^ while others pointing to the importance of adolescence.^27^ We make use of our multi-age cohorts to assess whether associations between poverty and health are age-sensitive. Fourth, we make use of longitudinal data to assess whether duration of time in poverty has an impact on hospitalisation outcomes, as has been suggested for other health outcomes.^27^ Fifth, our use of longitudinal data allows us to analyse within-individual change over an extended period - eight annual data collection waves - to estimate if associations are likely to be causal. Specifically, fixed effects regression analyses focus on within-individual change and control all ‘fixed’ differences (i.e., unchanging – or time invariant – confounding) between individuals that might otherwise explain associations between poverty and health.^16 28^”

*2) I believe the study would benefit from an upfront discussion on the potential pathways underlying the relationship between poverty (or deprivation in general) and child health, with a specific focus on the policy background in New Zealand. This should cover a discussion on access to quality health care as well as the affordability of such care. Other pathways, among many others, could be health behaviors and health education. Could it be that less wealthy parents are less aware of certain health risks and therefore are less likely to take their children to the hospital when they suffer certain conditions? Can they not afford to pay for certain treatments? In addition to including a section discussing potential mechanisms, I would recommend including directly testing for the role of some of these in the analysis if feasible in the data. This would help with comment 1 regarding the contributions of the study and could help tell a complete story explaining the (lack of) associations found in the main analysis.*

We thank the reviewer for this comment. We conceived of causal pathways in the design of this study but chose not to investigate them given there was little evidence of an association between poverty and the health outcomes we investigated. Specifically, we conceived of two potential pathways from poverty to health: lack of ‘economic investments’, and increased ‘family stress’. Taking on board the reviewers suggestion, we have tested these factors (described and shown at the end of this document). The results indicate little evidence that any of four factors – household crowding and food insecurity (as measures of economic investment) and parental smoking and parental psychological distress (as measures of family stress) – changed the associations between poverty and health. Moreover, only one factor – food insecurity – had any influence on the health outcomes we assessed. As such, and because describing the rationale, methods, results and discussing these findings takes up a lot of space, we do not believe that the addition of these results to the paper is justified. However, we are prepared to be guided by the editor and reviewer on this.

We have added the following paragraph to the introduction (lines 62-75) as an ‘*upfront discussion on the potential pathways underlying the relationship between poverty (or deprivation in general) and child health’:*

“A number of theories have been posited to explain the relationship between poverty and child health. One theory – the ‘economic investment’ theory – suggests that more affluent parents are better able to ‘invest’ more in their children’s development (e.g., through investments in nutrition, clothing, learning resources, housing, and healthcare) and this leads to better outcomes.^10^ Affluence can also overcome issues of access to health care, particularly if cost is a barrier, but even when it is not: e.g., New Zealand has universal health care with low co-payments in primary care, yet the odds of unmet need for primary health care is 1.4 times greater among those living in the most versus least deprived neighbourhoods.^11^ Another theory – the ‘family stress’ theory – suggests that living in poverty increases parental stress levels, and this stress hinders parents’ ability to provide quality care, which has negative impacts upon children’s outcomes.^12 13^ Other factors, such as health behaviours and health literacy may also play a role.^14 15^ Note that these factors are not mutually exclusive: the effect of poverty on children’s health could operate simultaneously through many pathways.”

*3) I agree that the use of longitudinal data provides benefits for the research question at hand. The paper mentions that the FE analysis are restricted to the subsample who have changes in recorded hospitalizations per year. In my understanding, you would also need variations in income/poverty status over time in this analysis. Can you provide evidence for the share of households in your sample that changes poverty status over time? If this share is pretty low, I believe this could explain the lack of significant association in the analysis. More information should be provided to increase the credibility of this model?*

Overall, 28.1% of households change poverty status at least once during the eight longitudinal waves so it was relatively common. We acknowledge this on p13, lines 295-297:

“Note, poverty status was variable across waves: 28.1% of children changed poverty status at least once across the eight waves.”

Other changes made

A few errors were noticed with Table 5 when putting together analyses for this response. First, a number of confidence intervals were calculated incorrectly. In most cases this was because +-1.96 multiplied by the standard error had been applied directly to the odds ratio, rather than being applied to the unexponentiated logit coefficients before exponentiating to give confidence intervals on the odds scale. These have now been calculated correctly and the Table and preceding text has been updated (line 384). Second, the coefficients for “Log of BHC income” were incorrectly labelled as β when they are odds ratios. As all coefficients in the Table are odds ratios, all within-table labels have been removed and “Panels show odd ratios (95% confidence intervals)” has been added to the Table legend. Third, the results for the quintiles of BHC income had been calculated using an incorrect variable. The table has now been updated with the correct results.

Also, Tony Blakely has asked to be removed as an author and to be acknowledged only. This has been updated in the manuscript and on the submission system (a change of authorship form has been included as part of the submissions).

Thank you for considering our revised manuscript.

Barry Milne, on behalf of all authors.

**Analysis of causal pathway mechanisms**

We investigated two possible theories explaining the relationship between poverty and child health: the *economic investment* theory and the *family stress* theory.(1) The economic investment theory suggests that more affluent parents are better able to ‘invest’ more in their children’s development (e.g., through investments in nutrition, clothing, learning resources, housing, and cultural activities) and this leads to better outcomes. That is, child poverty affects health through the indirect pathway of ‘investments’. Measure of ‘household crowding’ and ‘food insecurity’ are available in the IDI as indicators of (lack of) economic investment.

The family stress theory suggests that living in poverty increases parental stress levels, and this stress hinders parents’ ability to provide quality care, which has negative impacts upon children’s outcomes.(2,3) Thus, the family stress theory also suggests that poverty has an indirect effect on childhood outcomes through the influence that poverty has on parents. Measure of ‘parental smoking’ and ‘parental psychological distress’ are available in the IDI as indicators of family stress.

We will assess if there is evidence that lack of economic investment and family stress are causal pathways by which poverty impacts the health outcomes.

Economic investment measures

*Household crowding* was assessed using the Canadian National Occupancy Standard, which considers whether there is a deficit of bedrooms in a household given the number of occupants.(4,5) Household crowding was assessed at all waves in SOFIE as: 0 bedroom deficit (82.7-88.8% across waves), 1 bedroom deficit (6.8-10.1%), 2 bedroom deficit (1.1-2.4%), and 3+ bedroom deficit (0.1-0.5%). Across waves, 2.5-4.9% had missing data on this variable. Household crowding has been shown to elevate risk of hospitalisations for infectious disease.(6)

*Food insecurity* was assessed as endorsement by either parent of one or more of the following three items from the NZiDep,(7) assessed at waves 3, 5 and 7: (i) use of food grants or food banks; (ii) buying cheaper food so that other needed items can be bought; and (iii) going without fresh fruit and vegetables so that other needed items can be bought. 34.8%, 28.1%, and 38.9% of children were classified as food insecure, and 6.2%, 5.2% and 5.3% had missing data on this variable at waves 3, 5 and 7, respectively. Food insecurity has been shown to elevate risk of oral health conditions.(8)

Family stress measures

*Parental smoking* was assessed as current smoking among parents for assessment waves 3, 5 and 7. At waves 3, 5, and 7, 24.2%, 21.9%, and 18.8% of children had a mother who smoked, respectively, and 13.8%, 12.6%, and 10.8% had a father who smoked, respectively. For waves 3, 5 and 7, maternal data were unavailable for 8.6%, 10.3% , and 12.8% of children, respectively, and paternal data were unavailable for 34.4%, 35.4% and 37.6% of children, respectively. Note that a single parent family will necessarily have missing parental data, most commonly data for fathers. Parental smoking has been shown to elevate risk of child respiratory conditions and otitis media.(9,10)

*Parental psychological distress* was assessed using the Kessler-10, a scale measuring non-specific psychological distress.(11) Psychological distress was assessed for both parents at waves 3, 5 and 7 in SOFIE, as low (<=70^th^ percentile of scores), moderate (70^th^ - 90^th^ percentile of scores) and high (>90^th^ percentile of scores). For waves 3, 5 and 7, maternal data were unavailable for 9.0%, 10.6% , and 13.0% of children, respectively, and paternal data were unavailable for 34.8%, 35.6% and 37.8% of children, respectively. Parental mental health has been postulated as a potential mechanism mediating low income and childhood poor outcomes.(1)

Analysis

To assess whether the economic investment and family stress measures were on the causal pathway between childhood poverty and the hospitalisation outcomes, we reran the fixed effects and random effects models separately with the addition of each of the four economic investment and family stress measures. Evidence that a measure is on the causal (mediating) pathway would be shown if the association between poverty and an hospitalisation outcome is reduced by the introduction of the measure into the regression model. Thus, we compared the poverty coefficient before and after the (separate) introduction of each of the economic investment and family stress measures, for each hospitalisation outcome, and for each of the fixed effects and random effects models we computed. These models also allow an assessment of whether each of the measures has an independent association with the hospitalisation outcome. Note that we included a missing data category for each measure in the models so that analysis numbers were maintained.

Results

Fixed effects and random effects models for each hospitalisation outcome are shown in separate Tables, CP1-CP6. Each table has five columns, showing (i) the effect of poverty/income on the hospitalisation outcome without additional controls added (i.e., ‘unmediated’; these estimates are copied from Table 5 in the paper); and controlling for (ii) household crowding; (iii) food insecurity; (iv) parental smoking; and (v) parental psychological distress. Note that results for food insecurity, parental smoking and psychological distress are limited to three waves instead of the full eight as these measures were only assessed at waves 3, 5, and 7.

Results for all outcomes show no evidence that any factor changed the magnitude of association between child poverty and any hospitalization outcomes. For some models there were moderate changes in point estimates when measures of either food insecurity, parental smoking or parental psychological distress were included in models as mediators (e.g., fixed effect models for otitis media and oral health; random effect models of income quintiles for otitis media). In all such cases confidence intervals substantially overlapped between the mediated and unmediated models. Also, it should be noted that changes in estimates may be a function of the reduced analytic dataset (i.e., only three waves of data were analysed when food insecurity, parental smoking and parental psychological distress were assessed as causal pathways). Of the measures themselves, only food insecurity was shown to be associated with hospitalisation outcomes, particularly otitis media, infectious disease and preventable hospitalisations.

Discussion

There is no evidence that any of the economic investment and family stress measures tested were on the causal pathway between childhood poverty and the hospitalisation outcomes we investigated.

Citations

1. Cooper K, Stewart K. Does money affect children’s outcomes? An update. Case paper 2017;203.

2. Conger RD, Conger KJ, Martin MJ. Socioeconomic status, family processes, and individual development. Journal of Marriage and Family. 2010;72:685-704.

3. Conger RD, Ge X, Elder GH, Lorenz FO, Simons RL. Economic stress, coercive family process, and developmental problems of adolescents. Child development. 1994;65(2):541-61.

4. Pierse N, Carter K, Bierre S, Law D, Howden-Chapman P. Examining the role of tenure, household crowding and housing affordability on psychological distress, using longitudinal data. Journal of epidemiology and community health. 2016;70(10):961-6.

5. Statistics New Zealand. Crowded housing in New Zealand 1986-2006. Wellington: Statistics New Zealand; 2012.

6. Baker M, McDonald A, Zhang J, Howden-Chapman P. Infectious diseases attributable to household crowding in New Zealand: A systematic review and burden of disease estimate. Vol. 1. Wellington: He Kainga Oranga/Housing and Health Research; 2013.

7. Salmond C, King O, Crampton P, Waldegrave C. NZiDep: A New Zealand Index of Socioeconomic Deprivation for Individuals. Wellington: Dept of Public Health, University of Otago; 2005.

8. Mobley C, Marshall T, Milgrom P, Coldwell SE. The Contribution of Dietary Factors to Dental Caries and Disparities in Caries. Academic Pediatrics. 2009;9(6):410-14.

9. Byrnes CA, Trenholme A. Respiratory infections in Tamariki (children) and Taitamariki (young people) Māori, New Zealand. Journal of paediatrics and child health. 2010;46(9):521-6.

10. Håberg SE, Bentdal YE, London SJ, Kværner KJ, Nystad W, Nafstad P. Prenatal and postnatal parental smoking and acute otitis media in early childhood. Acta Paediatrica. 2010;99(1):99-105.

11. Kessler RC, Andrews G, Colpe LJ, Hiripi E, Mroczek DK, Normand S-L, et al. Short screening scales to monitor population prevalences and trends in non-specific psychological distress. Psychological medicine. 2002;32(06):959-76.

Table CP1. Longitudinal associations association between income (poverty) and otitis media hospitalisations in the SOFIE sample, with tests for different causal pathways. Panels show odd ratios (95% confidence intervals).

|  |  | **Causal pathways** | | | |
| --- | --- | --- | --- | --- | --- |
| **Fixed effect models** | Unmediated | Household Crowding^1^ | Food insecurity^2^ | Parental Smoking^3^ | Parental Psychological Distress^4^ |
| BHC income poverty | 0.75 (0.46-1.21) | 0.74 (0.46-1.21) | 0.86 (0.29-2.56) | 0.58 (0.17-1.94) | 0.83 (0.23-2.97) |
| Household Crowding^1^ |  |  |  |  |  |
| *0 bedroom deficit (ref)* |  |  |  |  |  |
| *1 bedroom deficit* |  | 0.65 (0.26-1.65) |  |  |  |
| *2 bedroom deficit* |  | 0.63 (0.06-6.58) |  |  |  |
| *3+ bedroom deficit* |  | 2.55 (0.25-25.66) |  |  |  |
| *Missing* |  | 1.69 (0.24-11.98) |  |  |  |
| Food insecurity^2^ |  |  |  |  |  |
| *Food secure (ref)* |  |  |  |  |  |
| *Food insecure* |  |  | 3.11 (1.00-9.65) |  |  |
| *Missing* |  |  | -- |  |  |
| Smoking^3^ |  |  |  |  |  |
| *Mother non-smoker (ref)* |  |  |  |  |  |
| *Mother current smoker* |  |  |  | 3.01 (0.23-39.40) |  |
| *Mother data missing* |  |  |  | -- |  |
| *Father non-smoker (ref)* |  |  |  |  |  |
| *Father current smoker* |  |  |  | 0.25 (0.01-6.60) |  |
| *Father data missing* |  |  |  | 10.39 (0.63-169.56) |  |
| Psychological Distress^4^ |  |  |  |  |  |
| *Mother low distress (ref)* |  |  |  |  |  |
| *Mother moderate distress* |  |  |  |  | 0.52 (0.11-2034) |
| *Mother high distress* |  |  |  |  | 0.37 (0.07-1.92) |
| *Mother data missing* |  |  |  |  | -- |
| *Father low distress (ref)* |  |  |  |  |  |
| *Father moderate distress* |  |  |  |  | 0.75 (0.06-9.45) |
| *Father high distress* |  |  |  |  | -- |
| *Father data missing* |  |  |  |  | 13.10 (0.75-227.63) |
| Number of children | 162 | 162 | 54 | 54 | 54 |
| Number of observations | 1,029 | 1,029 | 147 | 147 | 147 |

|  |  | **Causal pathways** | | | |
| --- | --- | --- | --- | --- | --- |
| **Fixed effect models** | Unmediated | Household Crowding^1^ | Food insecurity^2^ | Parental Smoking^3^ | Parental Psychological Distress^4^ |
| Log of BHC income | 1.44 (1.02-2.03) | 1.44 (1.02-2.04) | 1.00 (0.49-2.01) | 1.06 (0.51-2.22) | 0.94 (0.41-2.15) |
| Household Crowding^1^ |  |  |  |  |  |
| *0 bedroom deficit (ref)* |  |  |  |  |  |
| *1 bedroom deficit* |  | 0.66 (0.26-1.66) |  |  |  |
| *2 bedroom deficit* |  | 0.67 (0.07-6.91) |  |  |  |
| *3+ bedroom deficit* |  | 2.61 (0.27-25.65) |  |  |  |
| *Missing* |  | 1.86 (0.26-13.12) |  |  |  |
| Food insecurity^2^ |  |  |  |  |  |
| *Food secure (ref)* |  |  |  |  |  |
| *Food insecure* |  |  | 3.49 (1.07-11.31) |  |  |
| *Missing* |  |  | -- |  |  |
| Smoking^3^ |  |  |  |  |  |
| *Mother non-smoker (ref)* |  |  |  |  |  |
| *Mother current smoker* |  |  |  | 2.06 (0.18-24.05) |  |
| *Mother data missing* |  |  |  | -- |  |
| *Father non-smoker (ref)* |  |  |  |  |  |
| *Father current smoker* |  |  |  | 0.33 (0.01-12.64) |  |
| *Father data missing* |  |  |  | 7.01 (0.46-105.93) |  |
| Psychological Distress^4^ |  |  |  |  |  |
| *Mother low distress (ref)* |  |  |  |  |  |
| *Mother moderate distress* |  |  |  |  | 0.54 (0.11-2.52) |
| *Mother high distress* |  |  |  |  | 0.37 (0.01-1.90) |
| *Mother data missing* |  |  |  |  | -- |
| *Father low distress (ref)* |  |  |  |  |  |
| *Father moderate distress* |  |  |  |  | 0.81 (0.07-10.09) |
| *Father high distress* |  |  |  |  | -- |
| *Father data missing* |  |  |  |  | 10.27 (0.68-155.82) |
| Number of children | 162 | 162 | 54 | 54 | 54 |
| Number of observations | 1,029 | 1,029 | 147 | 147 | 147 |

|  |  | **Causal pathways** | | | |
| --- | --- | --- | --- | --- | --- |
| **Random intercepts models** | Unmediated | Household Crowding^1^ | Food insecurity^2^ | Parental Smoking^3^ | Parental Psychological Distress^4^ |
| BHC income poverty | 1.07 (0.76-1.52) | 1.09 (0.77-1.54) | 1.15 (0.65-2.05) | 1.39 (0.78-2.50) | 1.41 (0.79-2.55) |
| Household Crowding^1^ |  |  |  |  |  |
| *0 bedroom deficit (ref)* |  |  |  |  |  |
| *1 bedroom deficit* |  | 0.58 (0.30-1.11) |  |  |  |
| *2 bedroom deficit* |  | 1.15 (0.38-3.48) |  |  |  |
| *3+ bedroom deficit* |  | 2.50 (0.44-14.11) |  |  |  |
| *Missing* |  | 1.86 (0.57-6.04) |  |  |  |
| Food insecurity^2^ |  |  |  |  |  |
| *Food secure (ref)* |  |  |  |  |  |
| *Food insecure* |  |  | 2.20 (1.22-3.98) |  |  |
| *Missing* |  |  | 1.33 (0.40-4.46) |  |  |
| Smoking^3^ |  |  |  |  |  |
| *Mother non-smoker (ref)* |  |  |  |  |  |
| *Mother current smoker* |  |  |  | 0.84 (0.40-1.74) |  |
| *Mother data missing* |  |  |  | 0.76 (0.31-1.88) |  |
| *Father non-smoker (ref)* |  |  |  |  |  |
| *Father current smoker* |  |  |  | 0.95 (0.37-2.46) |  |
| *Father data missing* |  |  |  | 1.14 (0.58-2.21) |  |
| Psychological Distress^4^ |  |  |  |  |  |
| *Mother low distress (ref)* |  |  |  |  |  |
| *Mother moderate distress* |  |  |  |  | 0.84 (0.36-1.93) |
| *Mother high distress* |  |  |  |  | 1.66 (0.69-4.03) |
| *Mother data missing* |  |  |  |  | 0.80 (0.33-1.95) |
| *Father low distress (ref)* |  |  |  |  |  |
| *Father moderate distress* |  |  |  |  | 0.48 (0.13-1.72) |
| *Father high distress* |  |  |  |  | 0.26 (0.03-2.51) |
| *Father data missing* |  |  |  |  | 0.92 (0.49-1.73) |
| Number of children | 9,216 | 9,216 | 6,582 | 6,582 | 6,582 |
| Number of observations | 38,919 | 38,919 | 13,902 | 13,902 | 13,902 |

|  |  | **Causal pathways** | | | |
| --- | --- | --- | --- | --- | --- |
| **Random intercepts models** | Unmediated | Household Crowding^1^ | Food insecurity^2^ | Parental Smoking^3^ | Parental Psychological Distress^4^ |
| Log of BHC income | 0.91 (0.76-1.08) | 0.91 (0.76-1.08) | 0.85 (0.64-1.13) | 0.79 (0.60-1.04) | 0.78 (0.59-1.03) |
| Household Crowding^1^ |  |  |  |  |  |
| *0 bedroom deficit (ref)* |  |  |  |  |  |
| *1 bedroom deficit* |  | 0.58 (0.30-1.10) |  |  |  |
| *2 bedroom deficit* |  | 1.14 (0.38-3.45) |  |  |  |
| *3+ bedroom deficit* |  | 2.47 (0.44-13.87) |  |  |  |
| *Missing* |  | 1.79 (0.55-5.80) |  |  |  |
| Food insecurity^2^ |  |  |  |  |  |
| *Food secure (ref)* |  |  |  |  |  |
| *Food insecure* |  |  | 2.11 (1.18-3.80) |  |  |
| *Missing* |  |  | 1.28 (0.38-4.30) |  |  |
| Smoking^3^ |  |  |  |  |  |
| *Mother non-smoker (ref)* |  |  |  |  |  |
| *Mother current smoker* |  |  |  | 0.84 (0.40-1.75) |  |
| *Mother data missing* |  |  |  | 0.76 (0.31-1.89) |  |
| *Father non-smoker (ref)* |  |  |  |  |  |
| *Father current smoker* |  |  |  | 0.93 (0.36-2.42) |  |
| *Father data missing* |  |  |  | 1.10 (0.57-2.13) |  |
| Psychological Distress^4^ |  |  |  |  |  |
| *Mother low distress (ref)* |  |  |  |  |  |
| *Mother moderate distress* |  |  |  |  | 0.82 (0.36-1.90) |
| *Mother high distress* |  |  |  |  | 1.64 (0.68-3.98) |
| *Mother data missing* |  |  |  |  | 0.80 (0.33-1.94) |
| *Father low distress (ref)* |  |  |  |  |  |
| *Father moderate distress* |  |  |  |  | 0.47 (0.13-1.69) |
| *Father high distress* |  |  |  |  | 0.26 (0.03-2.49) |
| *Father data missing* |  |  |  |  | 0.90 (0.48-1.66) |
| Number of children | 9,216 | 9,216 | 6,582 | 6,582 | 6,582 |
| Number of observations | 38,919 | 38,919 | 13,902 | 13,902 | 13,902 |

|  |  | **Causal pathways** | | | |
| --- | --- | --- | --- | --- | --- |
| **Random intercepts models** | Unmediated | Household Crowding^1^ | Food insecurity^2^ | Parental Smoking^3^ | Parental Psychological Distress^4^ |
| **BHC income quintiles** |  |  |  |  |  |
| *1 (lowest 20%)* | 1.27 (0.71-2.27) | 1.31 (0.73-2.34) | 1.53 (0.56-4.16) | 2.18 (0.81-5.89) | 2.27 (0.84-6.15) |
| *2* | 1.48 (0.85-2.60) | 1.51 (0.86-2.63) | 1.45 (0.55-3.82) | 2.01 (0.77-5.21) | 2.03 (0.78-5.27) |
| *3* | 1.47 (0.85-2.55) | 1.49 (0.86-2.58) | 1.51 (0.58-3.92) | 1.85 (0.72-4.73) | 1.88 (0.73-4.81) |
| *4* | 0.91 (0.51-1.64) | 0.92 (0.51-1.65) | 1.09 (0.40-2.98) | 1.23 (0.45-3.35) | 1.22 (0.45-3.32) |
| *5 (highest 20%; ref)* |  |  |  |  |  |
| Household Crowding^1^ |  |  |  |  |  |
| *0 bedroom deficit (ref)* |  |  |  |  |  |
| *1 bedroom deficit* |  | 0.57 (0.30-1.09) |  |  |  |
| *2 bedroom deficit* |  | 1.14 (0.38-3.40) |  |  |  |
| *3+ bedroom deficit* |  | 2.39 (0.43-13.41) |  |  |  |
| *Missing* |  | 1.85 (0.57-5.99) |  |  |  |
| Food insecurity^2^ |  |  |  |  |  |
| *Food secure (ref)* |  |  |  |  |  |
| *Food insecure* |  |  | 2.07 (1.13-3.78) |  |  |
| *Missing* |  |  |  |  |  |
| Smoking^3^ |  |  |  |  |  |
| *Mother non-smoker (ref)* |  |  |  |  |  |
| *Mother current smoker* |  |  |  | 0.81 (0.39-1.68) |  |
| *Mother data missing* |  |  |  | 0.75 (0.31-1.85) |  |
| *Father non-smoker (ref)* |  |  |  |  |  |
| *Father current smoker* |  |  |  | 0.90 (0.35-2.33) |  |
| *Father data missing* |  |  |  | 1.07 (0.55-2.07) |  |
| Psychological Distress^4^ |  |  |  |  |  |
| *Mother low distress (ref)* |  |  |  |  |  |
| *Mother moderate distress* |  |  |  |  | 0.82 (0.36-1.89) |
| *Mother high distress* |  |  |  |  | 1.58 (0.65-3.82) |
| *Mother data missing* |  |  |  |  | 0.79 (0.33-1.92) |
| *Father low distress (ref)* |  |  |  |  |  |
| *Father moderate distress* |  |  |  |  | 0.45 (0.12-1.61) |
| *Father high distress* |  |  |  |  | 0.24 (0.02-2.36) |
| *Father data missing* |  |  |  |  | 0.86 (0.46-1.61) |
| Number of children | 9,216 | 9,216 | 6,582 | 6,582 | 6,582 |
| Number of observations | 38,919 | 38,919 | 13,902 | 13,902 | 13,902 |

Note: Fixed effect models use only the within-child variance and the estimate is an average of the estimated association between a change in poverty status, or a change in income on a change in the outcome for each included child. The number included in the fixed effect model is therefore small, as there must be a change in the outcome. Fixed effect models were adjusted for age, wave identifier, and the causal pathways in respective models. Random effect models were adjusted for age, age squared, ethnicity, and the causal pathways in respective models.

^1^Assessed at all waves using the Canadian National Occupancy Standard.

^2^Assessed at waves 3, 5, and 7 only using the NZiDep. Food insecurity was defined as endorsement by either parent of one or more of the following three items: (i) use of food grants or food banks; (ii) buying cheaper food so that other needed items can be bought; and (iii) going without fresh fruit and vegetables so that other needed items can be bought.

^3^Assessed at waves 3, 5, and 7 only.

^4^Assessed at waves 3, 5, and 7 only using the Kessler 10 scale. Low psychological distress was defined as scores of <5, moderate psychological distress as scores of 5-7, and high psychological distress as scores > 7.

Table CP2. Longitudinal associations association between income (poverty) and oral health hospitalisations in the SOFIE sample, with tests for different causal pathways. Panels show odd ratios (95% confidence intervals).

|  |  | **Causal pathways** | | | |
| --- | --- | --- | --- | --- | --- |
| **Fixed effect models** | Unmediated | Household Crowding^1^ | Food insecurity^2^ | Parental Smoking^3^ | Parental Psychological Distress^4^ |
| BHC income poverty | 0.49 (0.29-0.83) | 0.49 (0.29-0.83) | 0.89 (0.28-2.88) | 0.86 (0.29-2.56) | 1.23 (0.38-3.95) |
| Household Crowding^1^ |  |  |  |  |  |
| *0 bedroom deficit (ref)* |  |  |  |  |  |
| *1 bedroom deficit* |  | 0.72 (0.34-1.55) |  |  |  |
| *2 bedroom deficit* |  | 0.33 (0.05-2.23) |  |  |  |
| *3+ bedroom deficit* |  | 2.53 (0.07-95.2) |  |  |  |
| *Missing* |  | 2.99 (0.24-36.96) |  |  |  |
| Food insecurity^2^ |  |  |  |  |  |
| *Food secure (ref)* |  |  |  |  |  |
| *Food insecure* |  |  | 4.77 (1.44-15.86) |  |  |
| *Missing* |  |  | -- |  |  |
| Smoking^3^ |  |  |  |  |  |
| *Mother non-smoker (ref)* |  |  |  |  |  |
| *Mother current smoker* |  |  |  | 0.74 (0.13-4.11) |  |
| *Mother data missing* |  |  |  | -- |  |
| *Father non-smoker (ref)* |  |  |  |  |  |
| *Father current smoker* |  |  |  | 0.87 (0.07-10.12) |  |
| *Father data missing* |  |  |  | 1.15 (0.06-24.04) |  |
| Psychological Distress^4^ |  |  |  |  |  |
| *Mother low distress (ref)* |  |  |  |  |  |
| *Mother moderate distress* |  |  |  |  | 1.39 (0.46-4.23) |
| *Mother high distress* |  |  |  |  | 3.50 (0.56-21.70) |
| *Mother data missing* |  |  |  |  | -- |
| *Father low distress (ref)* |  |  |  |  |  |
| *Father moderate distress* |  |  |  |  | 1.23 (0.23-6.59) |
| *Father high distress* |  |  |  |  | -- |
| *Father data missing* |  |  |  |  | 1.40 (0.07-28.87) |
| Number of children | 162 | 162 | 51 | 51 | 51 |
| Number of observations | 1,023 | 1,023 | 144 | 144 | 144 |

|  |  | **Causal pathways** | | | |
| --- | --- | --- | --- | --- | --- |
| **Fixed effect models** | Unmediated | Household Crowding^1^ | Food insecurity^2^ | Parental Smoking^3^ | Parental Psychological Distress^4^ |
| Log of BHC income | 1.28 (0.87-1.90) | 1.28 (0.86-1.89) | 0.65 (0.28-1.50) | 0.54 (0.23-1.29) | 0.41 (0.15-1.08) |
| Household Crowding^1^ |  |  |  |  |  |
| *0 bedroom deficit (ref)* |  |  |  |  |  |
| *1 bedroom deficit* |  | 0.78 (0.37-1.66) |  |  |  |
| *2 bedroom deficit* |  | 0.34 (0.05-2.28) |  |  |  |
| *3+ bedroom deficit* |  | 2.69 (0.07-101.48) |  |  |  |
| *Missing* |  | 2.85 (0.23-34.88) |  |  |  |
| Food insecurity^2^ |  |  |  |  |  |
| *Food secure (ref)* |  |  |  |  |  |
| *Food insecure* |  |  | 4.71 (1.39-16.00) |  |  |
| *Missing* |  |  | -- |  |  |
| Smoking^3^ |  |  |  |  |  |
| *Mother non-smoker (ref)* |  |  |  |  |  |
| *Mother current smoker* |  |  |  | 0.76 (0.13-4.27) |  |
| *Mother data missing* |  |  |  | -- |  |
| *Father non-smoker (ref)* |  |  |  |  |  |
| *Father current smoker* |  |  |  | 0.73 (0.06-9.11) |  |
| *Father data missing* |  |  |  | 1.16 (0.05-24.58) |  |
| Psychological Distress^4^ |  |  |  |  |  |
| *Mother low distress (ref)* |  |  |  |  |  |
| *Mother moderate distress* |  |  |  |  | 1.50 (0.48-4.74) |
| *Mother high distress* |  |  |  |  | 5.49 (0.88-34.04) |
| *Mother data missing* |  |  |  |  | -- |
| *Father low distress (ref)* |  |  |  |  |  |
| *Father moderate distress* |  |  |  |  | 1.10 (0.21-5.89) |
| *Father high distress* |  |  |  |  | -- |
| *Father data missing* |  |  |  |  | 1.54 (0.07-32.10) |
| Number of children | 162 | 162 | 51 | 51 | 51 |
| Number of observations | 1,023 | 1,023 | 144 | 144 | 144 |

|  |  | **Causal pathways** | | | |
| --- | --- | --- | --- | --- | --- |
| **Random intercepts models** | Unmediated | Household Crowding^1^ | Food insecurity^2^ | Parental Smoking^3^ | Parental Psychological Distress^4^ |
| BHC income poverty | 0.96 (0.68-1.34) | 0.96 (0.68-1.34) | 0.85 (0.47-1.52) | 0.82 (0.45-1.48) | 0.83 (0.46-1.51) |
| Household Crowding^1^ |  |  |  |  |  |
| *0 bedroom deficit (ref)* |  |  |  |  |  |
| *1 bedroom deficit* |  | 1.08 (0.65-1.78) |  |  |  |
| *2 bedroom deficit* |  | 0.46 (0.10-2.03) |  |  |  |
| *3+ bedroom deficit* |  | 1.14 (0.14-9.48) |  |  |  |
| *Missing* |  | 1.06 (0.24-4.65) |  |  |  |
| Food insecurity^2^ |  |  |  |  |  |
| *Food secure (ref)* |  |  |  |  |  |
| *Food insecure* |  |  | 1.51 (0.86-2.65) |  |  |
| *Missing* |  |  | 0.60 (0.14-2.61) |  |  |
| Smoking^3^ |  |  |  |  |  |
| *Mother non-smoker (ref)* |  |  |  |  |  |
| *Mother current smoker* |  |  |  | 1.82 (0.96-3.43) |  |
| *Mother data missing* |  |  |  | 0.64 (0.22-1.87) |  |
| *Father non-smoker (ref)* |  |  |  |  |  |
| *Father current smoker* |  |  |  | 1.31 (0.57-2.97) |  |
| *Father data missing* |  |  |  | 1.26 (0.64-2.47) |  |
| Psychological Distress^4^ |  |  |  |  |  |
| *Mother low distress (ref)* |  |  |  |  |  |
| *Mother moderate distress* |  |  |  |  | 1.59 (0.80-3.14) |
| *Mother high distress* |  |  |  |  | 1.95 (0.86-4.45) |
| *Mother data missing* |  |  |  |  | 0.59 (0.20-1.71) |
| *Father low distress (ref)* |  |  |  |  |  |
| *Father moderate distress* |  |  |  |  | 0.83 (0.28-2.42) |
| *Father high distress* |  |  |  |  | 0.90 (0.20-4.01) |
| *Father data missing* |  |  |  |  | 1.24 (0.67-2.28) |
| Number of children | 9,216 | 9,216 | 6,582 | 6,582 | 6,582 |
| Number of observations | 38,919 | 38,919 | 13,902 | 13,902 | 13,902 |

|  |  | **Causal pathways** | | | |
| --- | --- | --- | --- | --- | --- |
| **Random intercepts models** | Unmediated | Household Crowding^1^ | Food insecurity^2^ | Parental Smoking^3^ | Parental Psychological Distress^4^ |
| Log of BHC income | 0.98 (0.82-1.17) | 0.98 (0.82-1.17) | 0.94 (0.70-1.28) | 0.96 (0.70-1.31) | 0.95 (0.70-1.29) |
| Household Crowding^1^ |  |  |  |  |  |
| *0 bedroom deficit (ref)* |  |  |  |  |  |
| *1 bedroom deficit* |  | 1.07 (0.65-1.76) |  |  |  |
| *2 bedroom deficit* |  | 0.45 (0.10-2.01) |  |  |  |
| *3+ bedroom deficit* |  | 1.13 (0.14-9.39) |  |  |  |
| *Missing* |  | 1.05 (0.24-4.60) |  |  |  |
| Food insecurity^2^ |  |  |  |  |  |
| *Food secure (ref)* |  |  |  |  |  |
| *Food insecure* |  |  | 1.41 (0.80-2.47) |  |  |
| *Missing* |  |  | 0.57 (0.13-2.49) |  |  |
| Smoking^3^ |  |  |  |  |  |
| *Mother non-smoker (ref)* |  |  |  |  |  |
| *Mother current smoker* |  |  |  | 1.78 (0.94-3.35) |  |
| *Mother data missing* |  |  |  | 0.64 (0.22-1.87) |  |
| *Father non-smoker (ref)* |  |  |  |  |  |
| *Father current smoker* |  |  |  | 1.30 (0.57-2.96) |  |
| *Father data missing* |  |  |  | 1.16 (0.59-2.27) |  |
| Psychological Distress^4^ |  |  |  |  |  |
| *Mother low distress (ref)* |  |  |  |  |  |
| *Mother moderate distress* |  |  |  |  | 1.56 (0.79-3.08) |
| *Mother high distress* |  |  |  |  | 1.90 (0.84-4.33) |
| *Mother data missing* |  |  |  |  | 0.59 (0.21-1.72) |
| *Father low distress (ref)* |  |  |  |  |  |
| *Father moderate distress* |  |  |  |  | 0.82 (0.28-2.40) |
| *Father high distress* |  |  |  |  | 0.87 (0.19-3.85) |
| *Father data missing* |  |  |  |  | 1.13 (0.61-2.08) |
| Number of children | 9,216 | 9,216 | 6,582 | 6,582 | 6,582 |
| Number of observations | 38,919 | 38,919 | 13,902 | 13,902 | 13,902 |

|  |  | **Causal pathways** | | | |
| --- | --- | --- | --- | --- | --- |
| **Random intercepts models** | Unmediated | Household Crowding^1^ | Food insecurity^2^ | Parental Smoking^3^ | Parental Psychological Distress^4^ |
| **BHC income quintiles** |  |  |  |  |  |
| *1 (lowest 20%)* | 1.59 (0.87-2.89) | 1.59 (0.87-2.90) | 1.56 (0.54-4.47) | 1.44 (0.50-4.14) | 1.56 (0.54-4.48) |
| *2* | 1.88 (1.06-3.35) | 1.90 (1.06-3.38) | 1.72 (0.63-4.70) | 1.61 (0.59-4.40) | 1.75 (0.64-4.76) |
| *3* | 1.88 (1.06-3.35) | 1.89 (1.06-3.37) | 1.77 (0.66-4.76) | 1.71 (0.64-4.57) | 1.82 (0.68-4.88) |
| *4* | 1.72 (0.95-3.09) | 1.73 (0.96-3.11) | 1.72 (0.63-4.66) | 1.66 (0.61-4.50) | 1.75 (0.65-4.74) |
| *5 (highest 20%; ref)* |  |  |  |  |  |
| Household Crowding^1^ |  |  |  |  |  |
| *0 bedroom deficit (ref)* |  |  |  |  |  |
| *1 bedroom deficit* |  | 1.04 (0.63-1.72) |  |  |  |
| *2 bedroom deficit* |  | 0.43 (0.10-1.92) |  |  |  |
| *3+ bedroom deficit* |  | 1.09 (0.13-9.07) |  |  |  |
| *Missing* |  | 1.04 (0.24-4.57) |  |  |  |
| Food insecurity^2^ |  |  |  |  |  |
| *Food secure (ref)* |  |  |  |  |  |
| *Food insecure* |  |  | 1.36 (0.77-2.41) |  |  |
| *Missing* |  |  | 0.57 (0.13-2.47) |  |  |
| Smoking^3^ |  |  |  |  |  |
| *Mother non-smoker (ref)* |  |  |  |  |  |
| *Mother current smoker* |  |  |  | 1.74 (0.92-3.29) |  |
| *Mother data missing* |  |  |  | 0.63 (0.21-1.84) |  |
| *Father non-smoker (ref)* |  |  |  |  |  |
| *Father current smoker* |  |  |  | 1.25 (0.55-2.84) |  |
| *Father data missing* |  |  |  | 1.16 (0.59-2.28) |  |
| Psychological Distress^4^ |  |  |  |  |  |
| *Mother low distress (ref)* |  |  |  |  |  |
| *Mother moderate distress* |  |  |  |  | 1.54 (0.78-3.05) |
| *Mother high distress* |  |  |  |  | 1.85 (0.81-4.23) |
| *Mother data missing* |  |  |  |  | 0.59 (0.20-1.70) |
| *Father low distress (ref)* |  |  |  |  |  |
| *Father moderate distress* |  |  |  |  | 0.80 (0.27-2.33) |
| *Father high distress* |  |  |  |  | 0.86 (0.19-3.81) |
| *Father data missing* |  |  |  |  | 1.12 (0.61-2.09) |
| Number of children | 9,216 | 9,216 | 6,582 | 6,582 | 6,582 |
| Number of observations | 38,919 | 38,919 | 13,902 | 13,902 | 13,902 |

Note: Fixed effect models use only the within-child variance and the estimate is an average of the estimated association between a change in poverty status, or a change in income on a change in the outcome for each included child. The number included in the fixed effect model is therefore small, as there must be a change in the outcome. Fixed effect models were adjusted for age, age squared, wave identifier, and the causal pathways in respective models. Random effect models were adjusted for age, age squared, ethnicity, and the causal pathways in respective models.

^1^Assessed at all waves using the Canadian National Occupancy Standard.

^2^Assessed at waves 3, 5, and 7 only using the NZiDep. Food insecurity was defined as endorsement by either parent of one or more of the following three items: (i) use of food grants or food banks; (ii) buying cheaper food so that other needed items can be bought; and (iii) going without fresh fruit and vegetables so that other needed items can be bought.

^3^Assessed at waves 3, 5, and 7 only.

^4^Assessed at waves 3, 5, and 7 only using the Kessler 10 scale. Low psychological distress was defined as scores of <5, moderate psychological distress as scores of 5-7, and high psychological distress as scores > 7.

Table CP3. Longitudinal associations association between income (poverty) and infectious disease hospitalisations in the SOFIE sample, with tests for different causal pathways. Panels show odd ratios (95% confidence intervals).

|  |  | **Causal pathways** | | | |
| --- | --- | --- | --- | --- | --- |
| **Fixed effect models** | Unmediated | Household Crowding^1^ | Food insecurity^2^ | Parental Smoking^3^ | Parental Psychological Distress^4^ |
| BHC income poverty | 0.82 (0.66-1.03) | 0.81 (0.65-1.01) | 0.75 (0.47-1.19) | 0.68 (0.42-1.08) | 0.65 (0.41-1.04) |
| Household Crowding^1^ |  |  |  |  |  |
| *0 bedroom deficit (ref)* |  |  |  |  |  |
| *1 bedroom deficit* |  | 0.89 (0.62-1.27) |  |  |  |
| *2 bedroom deficit* |  | 0.42 (0.19-0.91) |  |  |  |
| *3+ bedroom deficit* |  | 0.51 (0.11-2.35) |  |  |  |
| *Missing* |  | 0.50 (0.18-1.39) |  |  |  |
| Food insecurity^2^ |  |  |  |  |  |
| *Food secure (ref)* |  |  |  |  |  |
| *Food insecure* |  |  | 2.40 (1.53-3.78) |  |  |
| *Missing* |  |  | 6.65 (0.68-65.21) |  |  |
| Smoking^3^ |  |  |  |  |  |
| *Mother non-smoker (ref)* |  |  |  |  |  |
| *Mother current smoker* |  |  |  | 0.73 (0.31-1.70) |  |
| *Mother data missing* |  |  |  | 0.91 (0.11-7.50) |  |
| *Father non-smoker (ref)* |  |  |  |  |  |
| *Father current smoker* |  |  |  | 0.84 (0.26-2.68) |  |
| *Father data missing* |  |  |  | 4.28 (1.34-13.67) |  |
| Psychological Distress^4^ |  |  |  |  |  |
| *Mother low distress (ref)* |  |  |  |  |  |
| *Mother moderate distress* |  |  |  |  | 1.37 (0.80-2.34) |
| *Mother high distress* |  |  |  |  | 1.87 (0.89-3.94) |
| *Mother data missing* |  |  |  |  | 1.03 (0.13-8.24) |
| *Father low distress (ref)* |  |  |  |  |  |
| *Father moderate distress* |  |  |  |  | 0.49 (0.23-1.04) |
| *Father high distress* |  |  |  |  | 0.44 (0.12-1.69) |
| *Father data missing* |  |  |  |  | 4.09 (1.29-12.95) |
| Number of children | 873 | 873 | 279 | 279 | 279 |
| Number of observations | 5,103 | 5,103 | 750 | 750 | 750 |

|  |  | **Causal pathways** | | | |
| --- | --- | --- | --- | --- | --- |
| **Fixed effect models** | Unmediated | Household Crowding^1^ | Food insecurity^2^ | Parental Smoking^3^ | Parental Psychological Distress^4^ |
| Log of BHC income | 1.10 (0.96-1.26) | 1.10 (0.97-1.26) | 0.99 (0.74-1.32) | 1.02 (0.77-1.36) | 1.03 (0.77-1.37) |
| Household Crowding^1^ |  |  |  |  |  |
| *0 bedroom deficit (ref)* |  |  |  |  |  |
| *1 bedroom deficit* |  | 0.90 (0.63-1.28) |  |  |  |
| *2 bedroom deficit* |  | 0.43 (0.20-0.93) |  |  |  |
| *3+ bedroom deficit* |  | 0.52 (0.11-2.39) |  |  |  |
| *Missing* |  | 0.51 (0.18-1.42) |  |  |  |
| Food insecurity^2^ |  |  |  |  |  |
| *Food secure (ref)* |  |  |  |  |  |
| *Food insecure* |  |  | 2.42 (1.53-3.80) |  |  |
| *Missing* |  |  | 5.99 (0.59-60.91) |  |  |
| Smoking^3^ |  |  |  |  |  |
| *Mother non-smoker (ref)* |  |  |  |  |  |
| *Mother current smoker* |  |  |  | 0.69 (0.30-1.60) |  |
| *Mother data missing* |  |  |  | 0.78 (0.10-6.17) |  |
| *Father non-smoker (ref)* |  |  |  |  |  |
| *Father current smoker* |  |  |  | 0.79 (0.25-2.50) |  |
| *Father data missing* |  |  |  | 3.49 (1.12-10.86) |  |
| Psychological Distress^4^ |  |  |  |  |  |
| *Mother low distress (ref)* |  |  |  |  |  |
| *Mother moderate distress* |  |  |  |  | 1.37 (0.80-2.35) |
| *Mother high distress* |  |  |  |  | 1.89 (0.90-3.99) |
| *Mother data missing* |  |  |  |  | 0.91 (0.12-7.05) |
| *Father low distress (ref)* |  |  |  |  |  |
| *Father moderate distress* |  |  |  |  | 0.51 (0.24-1.08) |
| *Father high distress* |  |  |  |  | 0.43 (0.11-1.68) |
| *Father data missing* |  |  |  |  | 3.36 (1.09-10.40) |
| Number of children | 873 | 873 | 279 | 279 | 279 |
| Number of observations | 5,103 | 5,103 | 750 | 750 | 750 |

|  |  | **Causal pathways** | | | |
| --- | --- | --- | --- | --- | --- |
| **Random intercepts models** | Unmediated | Household Crowding^1^ | Food insecurity^2^ | Parental Smoking^3^ | Parental Psychological Distress^4^ |
| BHC income poverty | 1.04 (0.90-1.20) | 1.04 (0.90-1.20) | 0.88 (0.69-1.12) | 0.92 (0.72-1.18) | 0.92 (0.72-1.18) |
| Household Crowding^1^ |  |  |  |  |  |
| *0 bedroom deficit (ref)* |  |  |  |  |  |
| *1 bedroom deficit* |  | 0.93 (0.74-1.17) |  |  |  |
| *2 bedroom deficit* |  | 0.94 (0.59-1.50) |  |  |  |
| *3+ bedroom deficit* |  | 0.47 (0.14-1.62) |  |  |  |
| *Missing* |  | 1.02 (0.63-1.66) |  |  |  |
| Food insecurity^2^ |  |  |  |  |  |
| *Food secure (ref)* |  |  |  |  |  |
| *Food insecure* |  |  | 1.45 (1.14-1.84) |  |  |
| *Missing* |  |  | 1.31 (0.84-2.06) |  |  |
| Smoking^3^ |  |  |  |  |  |
| *Mother non-smoker (ref)* |  |  |  |  |  |
| *Mother current smoker* |  |  |  | 1.04 (0.78-1.39) |  |
| *Mother data missing* |  |  |  | 0.94 (0.67-1.32) |  |
| *Father non-smoker (ref)* |  |  |  |  |  |
| *Father current smoker* |  |  |  | 1.03 (0.72-1.48) |  |
| *Father data missing* |  |  |  | 1.16 (0.89-1.52) |  |
| Psychological Distress^4^ |  |  |  |  |  |
| *Mother low distress (ref)* |  |  |  |  |  |
| *Mother moderate distress* |  |  |  |  | 1.04 (0.76-1.44) |
| *Mother high distress* |  |  |  |  | 1.23 (0.82-1.83) |
| *Mother data missing* |  |  |  |  | 0.94 (0.67-1.31) |
| *Father low distress (ref)* |  |  |  |  |  |
| *Father moderate distress* |  |  |  |  | 0.76 (0.48-1.19) |
| *Father high distress* |  |  |  |  | 0.87 (0.45-0.70) |
| *Father data missing* |  |  |  |  | 1.09 (0.85-1.40) |
| Number of children | 9,216 | 9,216 | 6,582 | 6,582 | 6,582 |
| Number of observations | 38,919 | 38,919 | 13,902 | 13,902 | 13,902 |

|  |  | **Causal pathways** | | | |
| --- | --- | --- | --- | --- | --- |
| **Random intercepts models** | Unmediated | Household Crowding^1^ | Food insecurity^2^ | Parental Smoking^3^ | Parental Psychological Distress^4^ |
| Log of BHC income | 0.96 (0.89-1.03) | 0.96 (0.89-1.03) | 1.00 (0.88-1.12) | 0.97 (0.86-1.09) | 0.97 (0.86-1.09) |
| Household Crowding^1^ |  |  |  |  |  |
| *0 bedroom deficit (ref)* |  |  |  |  |  |
| *1 bedroom deficit* |  | 0.92 (0.73-1.17) |  |  |  |
| *2 bedroom deficit* |  | 0.94 (0.59-1.49) |  |  |  |
| *3+ bedroom deficit* |  | 0.47 (0.14-1.61) |  |  |  |
| *Missing* |  | 0.99 (0.61-1.60) |  |  |  |
| Food insecurity^2^ |  |  |  |  |  |
| *Food secure (ref)* |  |  |  |  |  |
| *Food insecure* |  |  | 1.40 (1.11-1.77) |  |  |
| *Missing* |  |  | 1.29 (0.82-2.02) |  |  |
| Smoking^3^ |  |  |  |  |  |
| *Mother non-smoker (ref)* |  |  |  |  |  |
| *Mother current smoker* |  |  |  | 1.03 (0.77-1.37) |  |
| *Mother data missing* |  |  |  | 0.94 (0.67-1.32) |  |
| *Father non-smoker (ref)* |  |  |  |  |  |
| *Father current smoker* |  |  |  | 1.03 (0.72-1.48) |  |
| *Father data missing* |  |  |  | 1.12 (0.86-1.46) |  |
| Psychological Distress^4^ |  |  |  |  |  |
| *Mother low distress (ref)* |  |  |  |  |  |
| *Mother moderate distress* |  |  |  |  | 1.04 (0.75-1.42) |
| *Mother high distress* |  |  |  |  | 1.21 (0.81-1.81) |
| *Mother data missing* |  |  |  |  | 0.94 (0.67-1.31) |
| *Father low distress (ref)* |  |  |  |  |  |
| *Father moderate distress* |  |  |  |  | 0.76 (0.48-1.19) |
| *Father high distress* |  |  |  |  | 0.86 (0.44-1.67) |
| *Father data missing* |  |  |  |  | 1.05 (0.81-1.34) |
| Number of children | 9,216 | 9,216 | 6,582 | 6,582 | 6,582 |
| Number of observations | 38,919 | 38,919 | 13,902 | 13,902 | 13,902 |

|  |  | **Causal pathways** | | | |
| --- | --- | --- | --- | --- | --- |
| **Random intercepts models** | Unmediated | Household Crowding^1^ | Food insecurity^2^ | Parental Smoking^3^ | Parental Psychological Distress^4^ |
| **BHC income quintiles** |  |  |  |  |  |
| *1 (lowest 20%)* | 1.26 (1.00-1.59) | 1.27 (1.01-1.60) | 0.94 (0.64-1.38) | 1.03 (0.70-1.52) | 1.05 (0.71-1.54) |
| *2* | 1.29 (1.03-1.61) | 1.30 (1.04-1.63) | 1.04 (0.72-1.50) | 1.15 (0.80-1.65) | 1.16 (0.81-1.67) |
| *3* | 1.37 (1.10-1.70) | 1.38 (1.10-1.71) | 1.29 (0.91-1.83) | 1.38 (0.97-1.94) | 1.40 (0.99-1.98) |
| *4* | 1.12 (0.89-1.41) | 1.12 (0.89-1.41) | 0.95 (0.66-1.37) | 0.98 (0.68-1.42) | 1.00 (0.69-1.44) |
| *5 (highest 20%; ref)* |  |  |  |  |  |
| Household Crowding^1^ |  |  |  |  |  |
| *0 bedroom deficit (ref)* |  |  |  |  |  |
| *1 bedroom deficit* |  | 0.91 (0.72-1.15) |  |  |  |
| *2 bedroom deficit* |  | 0.92 (0.58-1.47) |  |  |  |
| *3+ bedroom deficit* |  | 0.46 (0.14-1.57) |  |  |  |
| *Missing* |  | 1.01 (0.62-1.63) |  |  |  |
| Food insecurity^2^ |  |  |  |  |  |
| *Food secure (ref)* |  |  |  |  |  |
| *Food insecure* |  |  | 1.41 (1.10-1.79) |  |  |
| *Missing* |  |  | 1.29 (0.82-2.02) |  |  |
| Smoking^3^ |  |  |  |  |  |
| *Mother non-smoker (ref)* |  |  |  |  |  |
| *Mother current smoker* |  |  |  | 1.02 (0.76-1.37) |  |
| *Mother data missing* |  |  |  | 0.92 (0.66-1.30) |  |
| *Father non-smoker (ref)* |  |  |  |  |  |
| *Father current smoker* |  |  |  | 1.02 (0.71-1.47) |  |
| *Father data missing* |  |  |  | 1.13 (0.86-1.47) |  |
| Psychological Distress^4^ |  |  |  |  |  |
| *Mother low distress (ref)* |  |  |  |  |  |
| *Mother moderate distress* |  |  |  |  | 1.04 (0.75-1.43) |
| *Mother high distress* |  |  |  |  | 1.20 (0.80-1.79) |
| *Mother data missing* |  |  |  |  | 0.92 (0.66-1.29) |
| *Father low distress (ref)* |  |  |  |  |  |
| *Father moderate distress* |  |  |  |  | 0.73 (0.47-1.16) |
| *Father high distress* |  |  |  |  | 0.85 (0.44-1.66) |
| *Father data missing* |  |  |  |  | 1.05 (0.82-1.36) |
| Number of children | 9,216 | 9,216 | 6,582 | 6,582 | 6,582 |
| Number of observations | 38,919 | 38,919 | 13,902 | 13,902 | 13,902 |

Note: Fixed effect models use only the within-child variance and the estimate is an average of the estimated association between a change in poverty status, or a change in income on a change in the outcome for each included child. The number included in the fixed effect model is therefore small, as there must be a change in the outcome. Fixed effect models were adjusted for age, age squared, wave identifier, and the causal pathways in respective models. Random effect models were adjusted for age, age squared, ethnicity, and the causal pathways in respective models.

^1^Assessed at all waves using the Canadian National Occupancy Standard.

^2^Assessed at waves 3, 5, and 7 only using the NZiDep. Food insecurity was defined as endorsement by either parent of one or more of the following three items: (i) use of food grants or food banks; (ii) buying cheaper food so that other needed items can be bought; and (iii) going without fresh fruit and vegetables so that other needed items can be bought.

^3^Assessed at waves 3, 5, and 7 only.

^4^Assessed at waves 3, 5, and 7 only using the Kessler 10 scale. Low psychological distress was defined as scores of <5, moderate psychological distress as scores of 5-7, and high psychological distress as scores > 7.

Table CP4. Longitudinal associations association between income (poverty) and respiratory hospitalisations in the SOFIE sample, with tests for different causal pathways. Panels show odd ratios (95% confidence intervals).

|  |  | **Causal pathways** | | | |
| --- | --- | --- | --- | --- | --- |
| **Fixed effect models** | Unmediated | Household Crowding^1^ | Food insecurity^2^ | Parental Smoking^3^ | Parental Psychological Distress^4^ |
| BHC income poverty | 1.26 (0.80-2.00) | 1.26 (0.79-1.99) | 1.16 (0.45-2.97) | 0.85 (0.31-2.35) | 1.00 (0.35-2.85) |
| Household Crowding^1^ |  |  |  |  |  |
| *0 bedroom deficit (ref)* |  |  |  |  |  |
| *1 bedroom deficit* |  | 1.06 (0.52-2.15) |  |  |  |
| *2 bedroom deficit* |  | 1.06 (0.30-3.74) |  |  |  |
| *3+ bedroom deficit* |  | -- |  |  |  |
| *Missing* |  | 1.81 (0.26-12.90) |  |  |  |
| Food insecurity^2^ |  |  |  |  |  |
| *Food secure (ref)* |  |  |  |  |  |
| *Food insecure* |  |  | 1.14 (0.37-3.44) |  |  |
| *Missing* |  |  | -- |  |  |
| Smoking^3^ |  |  |  |  |  |
| *Mother non-smoker (ref)* |  |  |  |  |  |
| *Mother current smoker* |  |  |  | 2.91 (0.38-22.13) |  |
| *Mother data missing* |  |  |  | -- |  |
| *Father non-smoker (ref)* |  |  |  |  |  |
| *Father current smoker* |  |  |  | -- |  |
| *Father data missing* |  |  |  | -- |  |
| Psychological Distress^4^ |  |  |  |  |  |
| *Mother low distress (ref)* |  |  |  |  |  |
| *Mother moderate distress* |  |  |  |  | 1.40 (0.43-4.60) |
| *Mother high distress* |  |  |  |  | 0.27 (0.04-1.72) |
| *Mother data missing* |  |  |  |  | -- |
| *Father low distress (ref)* |  |  |  |  |  |
| *Father moderate distress* |  |  |  |  | 0.36 (0.08-1.65) |
| *Father high distress* |  |  |  |  | 0.23 (0.01-3.64) |
| *Father data missing* |  |  |  |  | 0.33 (0.01-7.67) |
| Number of children | 273 | 273 | 81 | 81 | 81 |
| Number of observations | 1,470 | 1,470 | 204 | 204 | 204 |

|  |  | **Causal pathways** | | | |
| --- | --- | --- | --- | --- | --- |
| **Fixed effect models** | Unmediated | Household Crowding^1^ | Food insecurity^2^ | Parental Smoking^3^ | Parental Psychological Distress^4^ |
| Log of BHC income | 0.91 (0.70-1.20) | 0.91 (0.69-1.20) | 1.07 (0.56-2.06) | 1.05 (0.56-1.94) | 1.09 (0.57-2.09) |
| Household Crowding^1^ |  |  |  |  |  |
| *0 bedroom deficit (ref)* |  |  |  |  |  |
| *1 bedroom deficit* |  | 1.04 (0.51-2.10) |  |  |  |
| *2 bedroom deficit* |  | 1.04 (0.29-3.67) |  |  |  |
| *3+ bedroom deficit* |  | -- |  |  |  |
| *Missing* |  | 1.90 (0.26-13.67) |  |  |  |
| Food insecurity^2^ |  |  |  |  |  |
| *Food secure (ref)* |  |  |  |  |  |
| *Food insecure* |  |  | 1.13 (0.37-3.44) |  |  |
| *Missing* |  |  | -- |  |  |
| Smoking^3^ |  |  |  |  |  |
| *Mother non-smoker (ref)* |  |  |  |  |  |
| *Mother current smoker* |  |  |  | 2.71 (0.38-19.43) |  |
| *Mother data missing* |  |  |  | -- |  |
| *Father non-smoker (ref)* |  |  |  |  |  |
| *Father current smoker* |  |  |  | -- |  |
| *Father data missing* |  |  |  | -- |  |
| Psychological Distress^4^ |  |  |  |  |  |
| *Mother low distress (ref)* |  |  |  |  |  |
| *Mother moderate distress* |  |  |  |  | 1.39 (0.43-4.46) |
| *Mother high distress* |  |  |  |  | 0.27 (0.04-1.65) |
| *Mother data missing* |  |  |  |  | -- |
| *Father low distress (ref)* |  |  |  |  |  |
| *Father moderate distress* |  |  |  |  | 0.35 (0.08-1.59) |
| *Father high distress* |  |  |  |  | 0.23 (0.01-3.64) |
| *Father data missing* |  |  |  |  | 0.33 (0.01-7.43) |
| Number of children | 273 | 273 | 81 | 81 | 81 |
| Number of observations | 1,470 | 1,470 | 204 | 204 | 204 |

|  |  | **Causal pathways** | | | |
| --- | --- | --- | --- | --- | --- |
| **Random intercepts models** | Unmediated | Household Crowding^1^ | Food insecurity^2^ | Parental Smoking^3^ | Parental Psychological Distress^4^ |
| BHC income poverty | 1.02 (0.78-1.34) | 1.02 (0.78-1.34) | 0.78 (0.49-1.23) | 0.89 (0.56-1.41) | 0.86 (0.54-1.38) |
| Household Crowding^1^ |  |  |  |  |  |
| *0 bedroom deficit (ref)* |  |  |  |  |  |
| *1 bedroom deficit* |  | 0.94 (0.62-1.42) |  |  |  |
| *2 bedroom deficit* |  | 0.99 (0.44-2.23) |  |  |  |
| *3+ bedroom deficit* |  | -- |  |  |  |
| *Missing* |  | 1.76 (0.78-4.00) |  |  |  |
| Food insecurity^2^ |  |  |  |  |  |
| *Food secure (ref)* |  |  |  |  |  |
| *Food insecure* |  |  | 1.47 (0.94-2.29) |  |  |
| *Missing* |  |  | 1.05 (0.45-2.45) |  |  |
| Smoking^3^ |  |  |  |  |  |
| *Mother non-smoker (ref)* |  |  |  |  |  |
| *Mother current smoker* |  |  |  | 0.78 (0.43-1.39) |  |
| *Mother data missing* |  |  |  | 0.69 (0.37-1.30) |  |
| *Father non-smoker (ref)* |  |  |  |  |  |
| *Father current smoker* |  |  |  | 0.87 (0.42-1.77) |  |
| *Father data missing* |  |  |  | 0.98 (0.59-1.62) |  |
| Psychological Distress^4^ |  |  |  |  |  |
| *Mother low distress (ref)* |  |  |  |  |  |
| *Mother moderate distress* |  |  |  |  | 1.22 (0.68-2.18) |
| *Mother high distress* |  |  |  |  | 1.25 (0.58-2.67) |
| *Mother data missing* |  |  |  |  | 0.77 (0.41-1.43) |
| *Father low distress (ref)* |  |  |  |  |  |
| *Father moderate distress* |  |  |  |  | 0.71 (0.30-1.64) |
| *Father high distress* |  |  |  |  | 0.62 (0.16-2.39) |
| *Father data missing* |  |  |  |  | 0.88 (0.54-1.42) |
| Number of children | 9,216 | 9,216 | 6,582 | 6,582 | 6,582 |
| Number of observations | 38,919 | 38,919 | 13,902 | 13,902 | 13,902 |

|  |  | **Causal pathways** | | | |
| --- | --- | --- | --- | --- | --- |
| **Random intercepts models** | Unmediated | Household Crowding^1^ | Food insecurity^2^ | Parental Smoking^3^ | Parental Psychological Distress^4^ |
| Log of BHC income | 1.01 (0.88-1.15) | 1.01 (0.88-1.16) | 1.02 (0.81-1.28) | 0.96 (0.77-1.20) | 0.97 (0.77-1.21) |
| Household Crowding^1^ |  |  |  |  |  |
| *0 bedroom deficit (ref)* |  |  |  |  |  |
| *1 bedroom deficit* |  | 0.94 (0.62-1.43) |  |  |  |
| *2 bedroom deficit* |  | 1.00 (0.44-2.24) |  |  |  |
| *3+ bedroom deficit* |  | -- |  |  |  |
| *Missing* |  | 1.78 (0.78-4.07) |  |  |  |
| Food insecurity^2^ |  |  |  |  |  |
| *Food secure (ref)* |  |  |  |  |  |
| *Food insecure* |  |  | 1.39 (0.89-2.17) |  |  |
| *Missing* |  |  | 1.03 (0.44-2.42) |  |  |
| Smoking^3^ |  |  |  |  |  |
| *Mother non-smoker (ref)* |  |  |  |  |  |
| *Mother current smoker* |  |  |  | 0.76 (0.42-1.36) |  |
| *Mother data missing* |  |  |  | 0.70 (0.37-1.30) |  |
| *Father non-smoker (ref)* |  |  |  |  |  |
| *Father current smoker* |  |  |  | 0.86 (0.42-1.77) |  |
| *Father data missing* |  |  |  | 0.93 (0.56-1.53) |  |
| Psychological Distress^4^ |  |  |  |  |  |
| *Mother low distress (ref)* |  |  |  |  |  |
| *Mother moderate distress* |  |  |  |  | 1.21 (0.68-2.16) |
| *Mother high distress* |  |  |  |  | 1.22 (0.57-2.61) |
| *Mother data missing* |  |  |  |  | 0.77 (0.41-1.44) |
| *Father low distress (ref)* |  |  |  |  |  |
| *Father moderate distress* |  |  |  |  | 0.70 (0.30-1.64) |
| *Father high distress* |  |  |  |  | 0.61 (0.16-2.34) |
| *Father data missing* |  |  |  |  | 0.82 (0.51-1.33) |
| Number of children | 9,216 | 9,216 | 6,582 | 6,582 | 6,582 |
| Number of observations | 38,919 | 38,919 | 13,902 | 13,902 | 13,902 |

|  |  | **Causal pathways** | | | |
| --- | --- | --- | --- | --- | --- |
| **Random intercepts models** | Unmediated | Household Crowding^1^ | Food insecurity^2^ | Parental Smoking^3^ | Parental Psychological Distress^4^ |
| **BHC income quintiles** |  |  |  |  |  |
| *1 (lowest 20%)* | 0.99 (0.65-1.51) | 0.99 (0.65-1.51) | 0.61 (0.30-1.23) | 0.79 (0.39-1.59) | 0.75 (0.37-1.52) |
| *2* | 0.97 (0.64-1.48) | 0.97 (0.64-1.48) | 0.72 (0.37-1.42) | 0.91 (0.47-1.77) | 0.87 (0.44-1.69) |
| *3* | 1.02 (0.68-1.53) | 1.02 (0.68-1.53) | 0.81 (0.42-1.54) | 0.94 (0.50-1.79) | 0.92 (0.44-1.76) |
| *4* | 0.97 (0.64-1.47) | 0.97 (0.64-1.47) | 0.74 (0.38-1.44) | 0.80 (0.41-1.55) | 0.78 (0.40-1.53) |
| *5 (highest 20%; ref)* |  |  |  |  |  |
| Household Crowding^1^ |  |  |  |  |  |
| *0 bedroom deficit (ref)* |  |  |  |  |  |
| *1 bedroom deficit* |  | 0.94 (0.62-1.42) |  |  |  |
| *2 bedroom deficit* |  | 0.99 (0.44-2.22) |  |  |  |
| *3+ bedroom deficit* |  | -- |  |  |  |
| *Missing* |  | 1.77 (0.78-2.23) |  |  |  |
| Food insecurity^2^ |  |  |  |  |  |
| *Food secure (ref)* |  |  |  |  |  |
| *Food insecure* |  |  | 1.52 (0.96-2.41) |  |  |
| *Missing* |  |  | 1.06 (0.45-2.49) |  |  |
| Smoking^3^ |  |  |  |  |  |
| *Mother non-smoker (ref)* |  |  |  |  |  |
| *Mother current smoker* |  |  |  | 0.77 (0.43-1.39) |  |
| *Mother data missing* |  |  |  | 0.69 (0.37-1.30) |  |
| *Father non-smoker (ref)* |  |  |  |  |  |
| *Father current smoker* |  |  |  | 0.88 (0.43-1.81) |  |
| *Father data missing* |  |  |  | 0.97 (0.58-1.62) |  |
| Psychological Distress^4^ |  |  |  |  |  |
| *Mother low distress (ref)* |  |  |  |  |  |
| *Mother moderate distress* |  |  |  |  | 1.23 (0.69-2.19) |
| *Mother high distress* |  |  |  |  | 1.24 (0.58-2.67) |
| *Mother data missing* |  |  |  |  | 0.77 (0.41-1.44) |
| *Father low distress (ref)* |  |  |  |  |  |
| *Father moderate distress* |  |  |  |  | 0.71 (0.30-1.67) |
| *Father high distress* |  |  |  |  | 0.63 (0.16-2.43) |
| *Father data missing* |  |  |  |  | 0.87 (0.53-1.43) |
| Number of children | 9,216 | 9,216 | 6,582 | 6,582 | 6,582 |
| Number of observations | 38,919 | 38,919 | 13,902 | 13,902 | 13,902 |

Note: Fixed effect models use only the within-child variance and the estimate is an average of the estimated association between a change in poverty status, or a change in income on a change in the outcome for each included child. The number included in the fixed effect model is therefore small, as there must be a change in the outcome. Fixed effect models were adjusted for age, age squared, wave identifier, and the causal pathways in respective models. Random effect models were adjusted for age, age squared, ethnicity, and the causal pathways in respective models.

^1^Assessed at all waves using the Canadian National Occupancy Standard.

^2^Assessed at waves 3, 5, and 7 only using the NZiDep. Food insecurity was defined as endorsement by either parent of one or more of the following three items: (i) use of food grants or food banks; (ii) buying cheaper food so that other needed items can be bought; and (iii) going without fresh fruit and vegetables so that other needed items can be bought.

^3^Assessed at waves 3, 5, and 7 only.

^4^Assessed at waves 3, 5, and 7 only using the Kessler 10 scale. Low psychological distress was defined as scores of <5, moderate psychological distress as scores of 5-7, and high psychological distress as scores > 7.

Table CP5. Longitudinal associations association between income (poverty) and preventable hospitalisations in the SOFIE sample, with tests for different causal pathways. Panels show odd ratios (95% confidence intervals).

|  |  | **Causal pathways** | | | |
| --- | --- | --- | --- | --- | --- |
| **Fixed effect models** | Unmediated | Household Crowding^1^ | Food insecurity^2^ | Parental Smoking^3^ | Parental Psychological Distress^4^ |
| BHC income poverty | 0.77 (0.62-0.97) | 0.76 (0.61-0.96) | 0.81 (0.50-1.30) | 0.81 (0.50-1.31) | 0.80 (0.49-1.30) |
| Household Crowding^1^ |  |  |  |  |  |
| *0 bedroom deficit (ref)* |  |  |  |  |  |
| *1 bedroom deficit* |  | 0.90 (0.63-1.27) |  |  |  |
| *2 bedroom deficit* |  | 0.47 (0.22-0.97) |  |  |  |
| *3+ bedroom deficit* |  | 0.62 (0.16-2.39) |  |  |  |
| *Missing* |  | 0.89 (0.33-2.35) |  |  |  |
| Food insecurity^2^ |  |  |  |  |  |
| *Food secure (ref)* |  |  |  |  |  |
| *Food insecure* |  |  | 2.17 (1.34-3.49) |  |  |
| *Missing* |  |  | -- |  |  |
| Smoking^3^ |  |  |  |  |  |
| *Mother non-smoker (ref)* |  |  |  |  |  |
| *Mother current smoker* |  |  |  | 1.14 (0.50-2.60) |  |
| *Mother data missing* |  |  |  | 1.07 (0.06-18.04) |  |
| *Father non-smoker (ref)* |  |  |  |  |  |
| *Father current smoker* |  |  |  | 0.55 (0.17-1.80) |  |
| *Father data missing* |  |  |  | 2.00 (0.59-6.78) |  |
| Psychological Distress^4^ |  |  |  |  |  |
| *Mother low distress (ref)* |  |  |  |  |  |
| *Mother moderate distress* |  |  |  |  | 1.56 (0.90-2.70) |
| *Mother high distress* |  |  |  |  | 2.09 (0.97-4.50) |
| *Mother data missing* |  |  |  |  | 1.23 (0.07-21.88) |
| *Father low distress (ref)* |  |  |  |  |  |
| *Father moderate distress* |  |  |  |  | 0.48 (0.22-1.08) |
| *Father high distress* |  |  |  |  | 0.44 (0.13-1.53) |
| *Father data missing* |  |  |  |  | 2.08 (0.62-6.95) |
| Number of children | 837 | 837 | 267 | 267 | 267 |
| Number of observations | 4,905 | 4,905 | 720 | 720 | 720 |

|  |  | **Causal pathways** | | | |
| --- | --- | --- | --- | --- | --- |
| **Fixed effect models** | Unmediated | Household Crowding^1^ | Food insecurity^2^ | Parental Smoking^3^ | Parental Psychological Distress^4^ |
| Log of BHC income | 1.10 (0.96-1.27) | 1.10 (0.96-1.27) | 0.97 (0.72-1.31) | 0.94 (0.70-1.27) | 0.94 (0.70-1.27) |
| Household Crowding^1^ |  |  |  |  |  |
| *0 bedroom deficit (ref)* |  |  |  |  |  |
| *1 bedroom deficit* |  | 0.91 (0.64-1.29) |  |  |  |
| *2 bedroom deficit* |  | 0.49 (0.23-1.00) |  |  |  |
| *3+ bedroom deficit* |  | 0.64 (0.17-2.48) |  |  |  |
| *Missing* |  | 0.91 (0.34-2.42) |  |  |  |
| Food insecurity^2^ |  |  |  |  |  |
| *Food secure (ref)* |  |  |  |  |  |
| *Food insecure* |  |  | 2.15 (1.33-3.46) |  |  |
| *Missing* |  |  | -- |  |  |
| Smoking^3^ |  |  |  |  |  |
| *Mother non-smoker (ref)* |  |  |  |  |  |
| *Mother current smoker* |  |  |  | 1.09 (0.48-2.50) |  |
| *Mother data missing* |  |  |  | 0.89 (0.05-15.11) |  |
| *Father non-smoker (ref)* |  |  |  |  |  |
| *Father current smoker* |  |  |  | 0.52 (0.16-1.71) |  |
| *Father data missing* |  |  |  | 1.72 (0.52-5.68) |  |
| Psychological Distress^4^ |  |  |  |  |  |
| *Mother low distress (ref)* |  |  |  |  |  |
| *Mother moderate distress* |  |  |  |  | 1.56 (0.90-2.69) |
| *Mother high distress* |  |  |  |  | 2.14 (0.99-4.62) |
| *Mother data missing* |  |  |  |  | 1.03 (0.06-18.61) |
| *Father low distress (ref)* |  |  |  |  |  |
| *Father moderate distress* |  |  |  |  | 0.49 (0.22-1.09) |
| *Father high distress* |  |  |  |  | 0.42 (0.12-1.51) |
| *Father data missing* |  |  |  |  | 1.88 (0.55-5.89) |
| Number of children | 837 | 837 | 267 | 267 | 267 |
| Number of observations | 4,905 | 4,905 | 720 | 720 | 720 |

|  |  | **Causal pathways** | | | |
| --- | --- | --- | --- | --- | --- |
| **Random intercepts models** | Unmediated | Household Crowding^1^ | Food insecurity^2^ | Parental Smoking^3^ | Parental Psychological Distress^4^ |
| BHC income poverty | 0.99 (0.86-1.15) | 1.00 (0.86-1.16) | 0.79 (0.61-1.03) | 0.85 (0.66-1.11) | 0.85 (0.65-1.10) |
| Household Crowding^1^ |  |  |  |  |  |
| *0 bedroom deficit (ref)* |  |  |  |  |  |
| *1 bedroom deficit* |  | 0.92 (0.73-1.17) |  |  |  |
| *2 bedroom deficit* |  | 0.94 (0.58-1.52) |  |  |  |
| *3+ bedroom deficit* |  | 0.62 (0.21-1.90) |  |  |  |
| *Missing* |  | 1.06 (0.63-1.80) |  |  |  |
| Food insecurity^2^ |  |  |  |  |  |
| *Food secure (ref)* |  |  |  |  |  |
| *Food insecure* |  |  | 1.53 (1.19-1.96) |  |  |
| *Missing* |  |  | 0.97 (0.58-1.62) |  |  |
| Smoking^3^ |  |  |  |  |  |
| *Mother non-smoker (ref)* |  |  |  |  |  |
| *Mother current smoker* |  |  |  | 1.13 (0.83-1.54) |  |
| *Mother data missing* |  |  |  | 0.84 (0.58-1.22) |  |
| *Father non-smoker (ref)* |  |  |  |  |  |
| *Father current smoker* |  |  |  | 0.94 (0.64-1.38) |  |
| *Father data missing* |  |  |  | 1.60 (0.80-1.41) |  |
| Psychological Distress^4^ |  |  |  |  |  |
| *Mother low distress (ref)* |  |  |  |  |  |
| *Mother moderate distress* |  |  |  |  | 1.19 (0.86-1.64) |
| *Mother high distress* |  |  |  |  | 1.48 (0.98-2.22) |
| *Mother data missing* |  |  |  |  | 0.86 (0.60-1.23) |
| *Father low distress (ref)* |  |  |  |  |  |
| *Father moderate distress* |  |  |  |  | 0.73 (0.45-1.16) |
| *Father high distress* |  |  |  |  | 0.97 (0.51-1.87) |
| *Father data missing* |  |  |  |  | 1.03 (0.78-1.34) |
| Number of children | 9,216 | 9,216 | 6,582 | 6,582 | 6,582 |
| Number of observations | 38,919 | 38,919 | 13,902 | 13,902 | 13,902 |

|  |  | **Causal pathways** | | | |
| --- | --- | --- | --- | --- | --- |
| **Random intercepts models** | Unmediated | Household Crowding^1^ | Food insecurity^2^ | Parental Smoking^3^ | Parental Psychological Distress^4^ |
| Log of BHC income | 0.96 (0.89-1.03) | 0.96 (0.89-1.03) | 1.01 (0.89-1.15) | 0.97 (0.86-1.11) | 0.98 (0.86-1.11) |
| Household Crowding^1^ |  |  |  |  |  |
| *0 bedroom deficit (ref)* |  |  |  |  |  |
| *1 bedroom deficit* |  | 0.92 (0.72-1.16) |  |  |  |
| *2 bedroom deficit* |  | 0.93 (0.58-1.50) |  |  |  |
| *3+ bedroom deficit* |  | 0.62 (0.20-1.88) |  |  |  |
| *Missing* |  | 1.02 (0.60-1.74) |  |  |  |
| Food insecurity^2^ |  |  |  |  |  |
| *Food secure (ref)* |  |  |  |  |  |
| *Food insecure* |  |  | 1.45 (1.13-1.86) |  |  |
| *Missing* |  |  | 0.95 (0.57-1.58) |  |  |
| Smoking^3^ |  |  |  |  |  |
| *Mother non-smoker (ref)* |  |  |  |  |  |
| *Mother current smoker* |  |  |  | 1.11 (0.82-1.51) |  |
| *Mother data missing* |  |  |  | 0.85 (0.59-1.22) |  |
| *Father non-smoker (ref)* |  |  |  |  |  |
| *Father current smoker* |  |  |  | 0.94 (0.64-1.38) |  |
| *Father data missing* |  |  |  | 1.00 (0.76-1.33) |  |
| Psychological Distress^4^ |  |  |  |  |  |
| *Mother low distress (ref)* |  |  |  |  |  |
| *Mother moderate distress* |  |  |  |  | 1.17 (0.84-1.62) |
| *Mother high distress* |  |  |  |  | 1.45 (0.96-2.18) |
| *Mother data missing* |  |  |  |  | 0.86 (0.60-1.23) |
| *Father low distress (ref)* |  |  |  |  |  |
| *Father moderate distress* |  |  |  |  | 0.72 (0.45-1.16) |
| *Father high distress* |  |  |  |  | 0.95 (0.49-1.82) |
| *Father data missing* |  |  |  |  | 0.97 (0.74-1.26) |
| Number of children | 9,216 | 9,216 | 6,582 | 6,582 | 6,582 |
| Number of observations | 38,919 | 38,919 | 13,902 | 13,902 | 13,902 |

|  |  | **Causal pathways** | | | |
| --- | --- | --- | --- | --- | --- |
| **Random intercepts models** | Unmediated | Household Crowding^1^ | Food insecurity^2^ | Parental Smoking^3^ | Parental Psychological Distress^4^ |
| **BHC income quintiles** |  |  |  |  |  |
| *1 (lowest 20%)* | 1.33 (1.04-1.69) | 1.34 (1.05-1.71) | 0.96 (0.64-1.45) | 1.11 (0.74-1.67) | 1.11 (0.74-1.67) |
| *2* | 1.31 (1.03-1.66) | 1.32 (1.04-1.67) | 0.97 (0.65-1.44) | 1.11 (0.75-1.65) | 1.10 (0.74-1.63) |
| *3* | 1.43 (1.13-1.81) | 1.44 (1.14-1.82) | 1.26 (0.87-1.84) | 1.38 (0.96-2.00) | 1.39 (0.96-2.02) |
| *4* | 1.24 (0.98-1.58) | 1.25 (0.98-1.58) | 1.21 (0.83-1.76) | 1.27 (0.87-1.85) | 1.27 (0.87-1.85) |
| *5 (highest 20%; ref)* |  |  |  |  |  |
| Household Crowding^1^ |  |  |  |  |  |
| *0 bedroom deficit (ref)* |  |  |  |  |  |
| *1 bedroom deficit* |  | 0.90 (0.71-1.15) |  |  |  |
| *2 bedroom deficit* |  | 0.92 (0.57-1.48) |  |  |  |
| *3+ bedroom deficit* |  | 0.61 (0.20-1.85) |  |  |  |
| *Missing* |  | 1.04 (0.61-1.75) |  |  |  |
| Food insecurity^2^ |  |  |  |  |  |
| *Food secure (ref)* |  |  |  |  |  |
| *Food insecure* |  |  | 1.49 (1.15-1.92) |  |  |
| *Missing* |  |  | 0.96 (0.57-1.60) |  |  |
| Smoking^3^ |  |  |  |  |  |
| *Mother non-smoker (ref)* |  |  |  |  |  |
| *Mother current smoker* |  |  |  | 1.11 (0.82-1.51) |  |
| *Mother data missing* |  |  |  | 0.84 (0.58-1.20) |  |
| *Father non-smoker (ref)* |  |  |  |  |  |
| *Father current smoker* |  |  |  | 0.92 (0.62-1.36) |  |
| *Father data missing* |  |  |  | 1.03 (0.77-1.37) |  |
| Psychological Distress^4^ |  |  |  |  |  |
| *Mother low distress (ref)* |  |  |  |  |  |
| *Mother moderate distress* |  |  |  |  | 1.18 (0.85-1.63) |
| *Mother high distress* |  |  |  |  | 1.46 (0.97-2.20) |
| *Mother data missing* |  |  |  |  | 0.85 (0.59-1.22) |
| *Father low distress (ref)* |  |  |  |  |  |
| *Father moderate distress* |  |  |  |  | 0.70 (0.44-1.13) |
| *Father high distress* |  |  |  |  | 0.95 (0.50-1.83) |
| *Father data missing* |  |  |  |  | 0.99 (0.76-1.31) |
| Number of children | 9,216 | 9,216 | 6,582 | 6,582 | 6,582 |
| Number of observations | 38,919 | 38,919 | 13,902 | 13,902 | 13,902 |

Note: Fixed effect models use only the within-child variance and the estimate is an average of the estimated association between a change in poverty status, or a change in income on a change in the outcome for each included child. The number included in the fixed effect model is therefore small, as there must be a change in the outcome. Fixed effect models were adjusted for age, age squared, wave identifier, and the causal pathways in respective models. Random effect models were adjusted for age, age squared, ethnicity, and the causal pathways in respective models.

^1^Assessed at all waves using the Canadian National Occupancy Standard.

^2^Assessed at waves 3, 5, and 7 only using the NZiDep. Food insecurity was defined as endorsement by either parent of one or more of the following three items: (i) use of food grants or food banks; (ii) buying cheaper food so that other needed items can be bought; and (iii) going without fresh fruit and vegetables so that other needed items can be bought.

^3^Assessed at waves 3, 5, and 7 only.

^4^Assessed at waves 3, 5, and 7 only using the Kessler 10 scale. Low psychological distress was defined as scores of <5, moderate psychological distress as scores of 5-7, and high psychological distress as scores > 7.

Table CP6. Longitudinal associations association between income (poverty) and any hospitalisation in the SOFIE sample, with tests for different causal pathways. Panels show odd ratios (95% confidence intervals).

|  |  | **Causal pathways** | | | |
| --- | --- | --- | --- | --- | --- |
| **Fixed effect models** | Unmediated | Household Crowding^1^ | Food insecurity^2^ | Parental Smoking^3^ | Parental Psychological Distress^4^ |
| BHC income poverty | 0.89 (0.76-1.03) | 0.88 (0.76-1.02) | 0.84 (0.62-1.13) | 0.84 (0.62-1.13) | 0.82 (0.61-1.11) |
| Household Crowding^1^ |  |  |  |  |  |
| *0 bedroom deficit (ref)* |  |  |  |  |  |
| *1 bedroom deficit* |  | 0.79 (0.62-1.00) |  |  |  |
| *2 bedroom deficit* |  | 0.76 (0.47-1.22) |  |  |  |
| *3+ bedroom deficit* |  | 0.92 (0.35-2.40) |  |  |  |
| *Missing* |  | 0.77 (0.42-1.43) |  |  |  |
| Food insecurity^2^ |  |  |  |  |  |
| *Food secure (ref)* |  |  |  |  |  |
| *Food insecure* |  |  | 1.19 (0.90-1.56) |  |  |
| *Missing* |  |  | 0.97 (0.27-3.55) |  |  |
| Smoking^3^ |  |  |  |  |  |
| *Mother non-smoker (ref)* |  |  |  |  |  |
| *Mother current smoker* |  |  |  | 0.82 (0.48-1.40) |  |
| *Mother data missing* |  |  |  | 0.57 (0.17-1.90) |  |
| *Father non-smoker (ref)* |  |  |  |  |  |
| *Father current smoker* |  |  |  | 0.78 (0.42-1.45) |  |
| *Father data missing* |  |  |  | 1.16 (0.63-2.13) |  |
| Psychological Distress^4^ |  |  |  |  |  |
| *Mother low distress (ref)* |  |  |  |  |  |
| *Mother moderate distress* |  |  |  |  | 1.28 (0.93-1.75) |
| *Mother high distress* |  |  |  |  | 0.90 (0.57-1.44) |
| *Mother data missing* |  |  |  |  | 0.53 (0.18-1.52) |
| *Father low distress (ref)* |  |  |  |  |  |
| *Father moderate distress* |  |  |  |  | 0.85 (0.56-1.29) |
| *Father high distress* |  |  |  |  | 0.84 (0.44-1.61) |
| *Father data missing* |  |  |  |  | 1.24 (0.69-2.22) |
| Number of children | 1,857 | 1,857 | 669 | 669 | 669 |
| Number of observations | 11,007 | 11,007 | 1,815 | 1,815 | 1,815 |

|  |  | **Causal pathways** | | | |
| --- | --- | --- | --- | --- | --- |
| **Fixed effect models** | Unmediated | Household Crowding^1^ | Food insecurity^2^ | Parental Smoking^3^ | Parental Psychological Distress^4^ |
| Log of BHC income | 1.00 (0.92-1.09) | 1.00 (0.92-1.09) | 0.97 (0.82-1.16) | 0.97 (0.82-1.16) | 0.99 (0.83-1.18) |
| Household Crowding^1^ |  |  |  |  |  |
| *0 bedroom deficit (ref)* |  |  |  |  |  |
| *1 bedroom deficit* |  | 0.80 (0.63-1.01) |  |  |  |
| *2 bedroom deficit* |  | 0.77 (0.48-1.23) |  |  |  |
| *3+ bedroom deficit* |  | 0.93 (0.35-2.45) |  |  |  |
| *Missing* |  | 0.77 (0.42-1.43) |  |  |  |
| Food insecurity^2^ |  |  |  |  |  |
| *Food secure (ref)* |  |  |  |  |  |
| *Food insecure* |  |  | 1.18 (0.90-1.56) |  |  |
| *Missing* |  |  | 0.92 (0.25-3.36) |  |  |
| Smoking^3^ |  |  |  |  |  |
| *Mother non-smoker (ref)* |  |  |  |  |  |
| *Mother current smoker* |  |  |  | 0.81 (0.48-1.39) |  |
| *Mother data missing* |  |  |  | 0.53 (0.16-1.74) |  |
| *Father non-smoker (ref)* |  |  |  |  |  |
| *Father current smoker* |  |  |  | 0.77 (0.42-1.43) |  |
| *Father data missing* |  |  |  | 1.08 (0.59-1.96) |  |
| Psychological Distress^4^ |  |  |  |  |  |
| *Mother low distress (ref)* |  |  |  |  |  |
| *Mother moderate distress* |  |  |  |  | 1.27 (0.92-1.74) |
| *Mother high distress* |  |  |  |  | 0.92 (0.58-1.46) |
| *Mother data missing* |  |  |  |  | 0.50 (0.17-1.41) |
| *Father low distress (ref)* |  |  |  |  |  |
| *Father moderate distress* |  |  |  |  | 0.85 (0.56-1.29) |
| *Father high distress* |  |  |  |  | 0.83 (0.43-1.61) |
| *Father data missing* |  |  |  |  | 1.15 (0.65-2.05) |
| Number of children | 1,857 | 1,857 | 669 | 669 | 669 |
| Number of observations | 11,007 | 11,007 | 1,815 | 1,815 | 1,815 |

|  |  | **Causal pathways** | | | |
| --- | --- | --- | --- | --- | --- |
| **Random intercepts models** | Unmediated | Household Crowding^1^ | Food insecurity^2^ | Parental Smoking^3^ | Parental Psychological Distress^4^ |
| BHC income poverty | 1.01 (0.92-1.12) | 1.02 (0.92-1.12) | 0.86 (0.73-1.02) | 0.87 (0.73-1.03) | 0.87 (0.73-1.03) |
| Household Crowding^1^ |  |  |  |  |  |
| *0 bedroom deficit (ref)* |  |  |  |  |  |
| *1 bedroom deficit* |  | 0.94 (0.80-1.11) |  |  |  |
| *2 bedroom deficit* |  | 0.13 (0.82-1.56) |  |  |  |
| *3+ bedroom deficit* |  | 0.83 (0.40-1.70) |  |  |  |
| *Missing* |  | 0.95 (0.71-1.29) |  |  |  |
| Food insecurity^2^ |  |  |  |  |  |
| *Food secure (ref)* |  |  |  |  |  |
| *Food insecure* |  |  | 1.24 (1.05-1.45) |  |  |
| *Missing* |  |  | 1.13 (0.82-1.56) |  |  |
| Smoking^3^ |  |  |  |  |  |
| *Mother non-smoker (ref)* |  |  |  |  |  |
| *Mother current smoker* |  |  |  | 1.08 (0.89-1.32) |  |
| *Mother data missing* |  |  |  | 0.93 (0.72-1.18) |  |
| *Father non-smoker (ref)* |  |  |  |  |  |
| *Father current smoker* |  |  |  | 1.06 (0.84-1.36) |  |
| *Father data missing* |  |  |  | 1.13 (0.94-1.35) |  |
| Psychological Distress^4^ |  |  |  |  |  |
| *Mother low distress (ref)* |  |  |  |  |  |
| *Mother moderate distress* |  |  |  |  | 1.18 (0.96-1.45) |
| *Mother high distress* |  |  |  |  | 1.10 (0.83-1.46) |
| *Mother data missing* |  |  |  |  | 0.93 (0.73-1.18) |
| *Father low distress (ref)* |  |  |  |  |  |
| *Father moderate distress* |  |  |  |  | 0.95 (0.72-1.25) |
| *Father high distress* |  |  |  |  | 1.08 (0.71-1.65) |
| *Father data missing* |  |  |  |  | 1.11 (0.93-1.32) |
| Number of children | 9,216 | 9,216 | 6,582 | 6,582 | 6,582 |
| Number of observations | 38,919 | 38,919 | 13,902 | 13,902 | 13,902 |

|  |  | **Causal pathways** | | | |
| --- | --- | --- | --- | --- | --- |
| **Random intercepts models** | Unmediated | Household Crowding^1^ | Food insecurity^2^ | Parental Smoking^3^ | Parental Psychological Distress^4^ |
| Log of BHC income | 0.95 (0.91-1.00) | 0.95 (0.90-1.00) | 1.00 (0.93-1.09) | 1.00 (0.92-1.08) | 1.00 (0.92-1.08) |
| Household Crowding^1^ |  |  |  |  |  |
| *0 bedroom deficit (ref)* |  |  |  |  |  |
| *1 bedroom deficit* |  | 0.93 (0.79-1.09) |  |  |  |
| *2 bedroom deficit* |  | 1.12 (0.81-1.54) |  |  |  |
| *3+ bedroom deficit* |  | 0.82 (0.40-1.68) |  |  |  |
| *Missing* |  | 0.90 (0.66-1.22) |  |  |  |
| Food insecurity^2^ |  |  |  |  |  |
| *Food secure (ref)* |  |  |  |  |  |
| *Food insecure* |  |  | 1.20 (1.02-1.40) |  |  |
| *Missing* |  |  | 1.11 (0.80-1.53) |  |  |
| Smoking^3^ |  |  |  |  |  |
| *Mother non-smoker (ref)* |  |  |  |  |  |
| *Mother current smoker* |  |  |  | 1.07 (0.88-1.30) |  |
| *Mother data missing* |  |  |  | 0.93 (0.72-1.19) |  |
| *Father non-smoker (ref)* |  |  |  |  |  |
| *Father current smoker* |  |  |  | 1.06 (0.83-1.35) |  |
| *Father data missing* |  |  |  | 1.08 (0.91-1.30) |  |
| Psychological Distress^4^ |  |  |  |  |  |
| *Mother low distress (ref)* |  |  |  |  |  |
| *Mother moderate distress* |  |  |  |  | 1.17 (0.95-1.44) |
| *Mother high distress* |  |  |  |  | 1.09 (0.82-1.44) |
| *Mother data missing* |  |  |  |  | 0.93 (0.73-1.18) |
| *Father low distress (ref)* |  |  |  |  |  |
| *Father moderate distress* |  |  |  |  | 0.95 (0.72-1.25) |
| *Father high distress* |  |  |  |  | 1.06 (0.70-1.61) |
| *Father data missing* |  |  |  |  | 1.06 (0.90-1.26) |
| Number of children | 9,216 | 9,216 | 6,582 | 6,582 | 6,582 |
| Number of observations | 38,919 | 38,919 | 13,902 | 13,902 | 13,902 |

|  |  | **Causal pathways** | | | |
| --- | --- | --- | --- | --- | --- |
| **Random intercepts models** | Unmediated | Household Crowding^1^ | Food insecurity^2^ | Parental Smoking^3^ | Parental Psychological Distress^4^ |
| **BHC income quintiles** |  |  |  |  |  |
| *1 (lowest 20%)* | 1.19 (1.02-1.39) | 1.20 (1.02-1.40) | 0.97 (0.75-1.25) | 0.99 (0.77-1.29) | 1.00 (0.77-1.30) |
| *2* | 1.29 (1.11-1.50) | 1.29 (1.11-1.50) | 1.09 (0.85-1.39) | 1.12 (0.88-1.43) | 1.13 (0.89-1.44) |
| *3* | 1.31 (1.13-1.51) | 1.31 (1.13-1.52) | 1.33 (1.06-1.67) | 1.36 (1.08-1.70) | 1.37 (1.09-1.72) |
| *4* | 1.12 (0.96-1.30) | 1.12 (0.97-1.30) | 1.08 (0.86-1.36) | 1.09 (0.86-1.37) | 1.09 (0.87-1.38) |
| *5 (highest 20%; ref)* |  |  |  |  |  |
| Household Crowding^1^ |  |  |  |  |  |
| *0 bedroom deficit (ref)* |  |  |  |  |  |
| *1 bedroom deficit* |  | 0.92 (0.78-1.09) |  |  |  |
| *2 bedroom deficit* |  | 1.10 (0.80-1.52) |  |  |  |
| *3+ bedroom deficit* |  | 0.81 (0.40-1.66) |  |  |  |
| *Missing* |  | 0.95 (0.70-1.28) |  |  |  |
| Food insecurity^2^ |  |  |  |  |  |
| *Food secure (ref)* |  |  |  |  |  |
| *Food insecure* |  |  | 1.19 (1.01-1.40) |  |  |
| *Missing* |  |  | 1.10 (0.80-1.53) |  |  |
| Smoking^3^ |  |  |  |  |  |
| *Mother non-smoker (ref)* |  |  |  |  |  |
| *Mother current smoker* |  |  |  | 1.07 (0.88-1.30) |  |
| *Mother data missing* |  |  |  | 0.91 (0.71-1.17) |  |
| *Father non-smoker (ref)* |  |  |  |  |  |
| *Father current smoker* |  |  |  | 1.04 (0.82-1.33) |  |
| *Father data missing* |  |  |  | 1.09 (0.91-1.31) |  |
| Psychological Distress^4^ |  |  |  |  |  |
| *Mother low distress (ref)* |  |  |  |  |  |
| *Mother moderate distress* |  |  |  |  | 1.17 (0.95-1.44) |
| *Mother high distress* |  |  |  |  | 1.08 (0.81-1.43) |
| *Mother data missing* |  |  |  |  | 0.92 (0.72-1.17) |
| *Father low distress (ref)* |  |  |  |  |  |
| *Father moderate distress* |  |  |  |  | 0.92 (0.70-1.22) |
| *Father high distress* |  |  |  |  | 1.06 (0.70-1.61) |
| *Father data missing* |  |  |  |  | 1.07 (0.90-1.27) |
| Number of children | 9,216 | 9,216 | 6,582 | 6,582 | 6,582 |
| Number of observations | 38,919 | 38,919 | 13,902 | 13,902 | 13,902 |

Note: Fixed effect models use only the within-child variance and the estimate is an average of the estimated association between a change in poverty status, or a change in income on a change in the outcome for each included child. The number included in the fixed effect model is therefore small, as there must be a change in the outcome. Fixed effect models were adjusted for age, age squared, wave identifier, and the causal pathways in respective models. Random effect models were adjusted for age, age squared, ethnicity, and the causal pathways in respective models.

^1^Assessed at all waves using the Canadian National Occupancy Standard.

^2^Assessed at waves 3, 5, and 7 only using the NZiDep. Food insecurity was defined as endorsement by either parent of one or more of the following three items: (i) use of food grants or food banks; (ii) buying cheaper food so that other needed items can be bought; and (iii) going without fresh fruit and vegetables so that other needed items can be bought.

^3^Assessed at waves 3, 5, and 7 only.

^4^Assessed at waves 3, 5, and 7 only using the Kessler 10 scale. Low psychological distress was defined as scores of <5, moderate psychological distress as scores of 5-7, and high psychological distress as scores > 7.
